# Wearable Fabric Electrotactile System with Stimulation–Inhibition Electrode Units

**DOI:** 10.34133/cbsystems.0515

**Published:** 2026-04-01

**Authors:** Hongbo Yao, Delong Li, Wenjun Zhang, Qiwei Xiong, Yuhe Luo, Chuhang Lin, Jiyu Wang, Jialong Liu, Mingyu Tan, Xijie Wu, Yuanjun Ma, Yihuan Lin, Qingao Hu, Tao Huang, Lin Shu, Lei Wei, Xinge Yu, Xiangmin Xu

**Affiliations:** ^1^ School of Future Technology, South China University of Technology, Guangzhou 511442, China.; ^2^ School of Electronic and Information Engineering, South China University of Technology, Guangzhou 510641, China.; ^3^ Deakin University, Geelong 3217, Australia.; ^4^ Department of Biomedical Engineering, City University of Hong Kong, Hong Kong 999077, China.

## Abstract

Tactile feedback is crucial for enhancing the virtual-reality (VR) interaction experience. However, current electrotactile devices suffer from issues such as current diffusion and electrode crosstalk, limiting spatial accuracy. To address this challenge, we designed a fabric-based ultrathin flexible microelectrode array with novel stimulation–inhibition electrode units that reduces current diffusion and improves focusing, improving tactile feedback accuracy and clarity. Additionally, we developed an electrical tactile interaction evaluation system to quantitatively assess the tactile recognition accuracy and reaction time of 30 participants. Experimental results demonstrate that the proposed electrode structure and evaluation system substantially enhance tactile perception in VR environments. This system has been demonstrated through immersive scenarios such as touching running water, stroking a bird’s forehead, and feeling a cactus, highlighting its potential for providing precise tactile feedback and enhancing personalized human–computer interaction in VR.

## Introduction

The sense of touch, as 1 of the 5 senses, is an important means of perception for humans to interact with the external environment [1–3]. It enables us to discern the shape, texture, and physical properties of objects while also facilitating haptic cognition and exploration of the physical world. With the development of technologies such as virtual reality (VR) and augmented reality (AR), the interactive experience of the digital world has become increasingly rich [4–6]. However, despite these advancements, most digital interactions remain heavily reliant on vision and hearing, with tactile feedback often being overlooked or underdeveloped [7–9]. This lack of tactile engagement substantially limits user immersion and interaction accuracy in virtual environments, hindering the full potential of VR and AR applications.

In recent years, many studies have been devoted to the integration of tactile feedback into virtual experiences [4,10–15]. Electrotactile stimulation is ideal for tactile feedback in virtual environments due to its precise control of intensity, frequency, and duration [16–18]. Concurrently, recent advances in tactile interfaces—spanning self-powered multimodal skins that fuse triboelectric and magnetoelastic sensing, vision-integrated soft skins for high-resolution contact mapping, and bimodal “tactile tomography” for subsurface feature reconstruction—have pushed toward localized, high-fidelity skin–device interaction [19–21]. These developments further motivate the need for spatially precise, targeted electrotactile feedback. However, existing electrotactile technologies still face many challenges. For instance, although virtual tactile generation technology has been greatly developed, the improvement of tactile simulation and perception is still a technical challenge that needs to be broken through [22]. Initially, interference between electrodes substantially affects the accuracy of tactile perception, especially in multielectrode arrays, where current diffusion from neighboring electrodes leads to blurring of perception and reduces the user’s tactile discriminative ability. Furthermore, most existing tactile assessment methods rely on the traditional physical tests or scale assessment, which lacks dynamic, individualized training paradigms to meet personalized rehabilitation needs [23–27].

Several studies have focused on optimizing electrode parameters for specific applications, such as clinical assessments and human–machine interface (HMI) compatibility. For instance, Poulsen et al. [28] optimized electrode design parameters to preferentially activate small nerve fibers, facilitating clinical assessments of neuropathic pain. In parallel, Yang et al. [29] introduced a concentric-ring electrode design; in Yang et al.’s architecture, the inner and outer rings are driven with opposite polarities so that far-field potentials cancel, thereby reducing stimulation artifacts in simultaneously recorded electromyography and improving bidirectional HMI compatibility. However, this far-field suppression is not designed to address the near-field lateral current spread between adjacent pads that causes channel-to-channel crosstalk during electrotactile rendering, and it does not evaluate tactile perception outcomes on multielectrode arrays. Moreover, the guard-ring layout typically consumes area, which can trade off channel density and scalability in dense arrays. This limitation is particularly critical in VR environments, where precise tactile feedback with accurate spatial localization is essential. Accordingly, a gap remains for dense electrotactile arrays: an electrode strategy that effectively suppresses adjacent-electrode crosstalk and is validated on human perceptual metrics.

To address these challenges, we propose a fabric-based microelectrode array (FMA) incorporating a stimulation–inhibition electrode unit structure, aiming to improve the precision of tactile perception, especially for applications in VR environments. In contrast to conventional electrode designs, our stimulation–inhibition electrode unit substantially reduces interelectrode interference by inhibiting the current diffusion of the surrounding electrodes, thus improving the tactile perception effect of the center electrode. This design not only solves the problem of ambiguous tactile perception in multielectrode arrays but also enhances tactile recognition performance for VR-based tactile evaluation and training. Based on this electrode structure, we further developed a tactile perception evaluation interaction system (TPEIS) integrating a programmable electrical stimulation generator and a VR interface with an embedded evaluation protocol. This system supports a variety of tactile tasks in virtual environments and generates comprehensive metrics for personalized tactile training. Compared to traditional methods such as scale-based assessments or abrasive-material-testing protocols, the system offers greater convenience, quantitative precision in tactile perception evaluation, cost-effectiveness, and compatibility with diverse virtual training scenarios. The technological framework holds significant potential for implementation across multiple domains, including medical rehabilitation and VR and AR immersive experiences, while providing a noninvasive, safety-optimized training platform for populations exhibiting reduced tactile sensitivity. While the system does not directly restore tactile perception capabilities, it facilitates enhanced tactile sensitivity within immersive virtual environments through the implementation of precisely calibrated tactile perception tasks and virtual training protocols, thereby enabling rigorous quantitative assessment methodologies.

## Materials and Methods

### The design scheme of the TPEIS

**Fig. 1.**
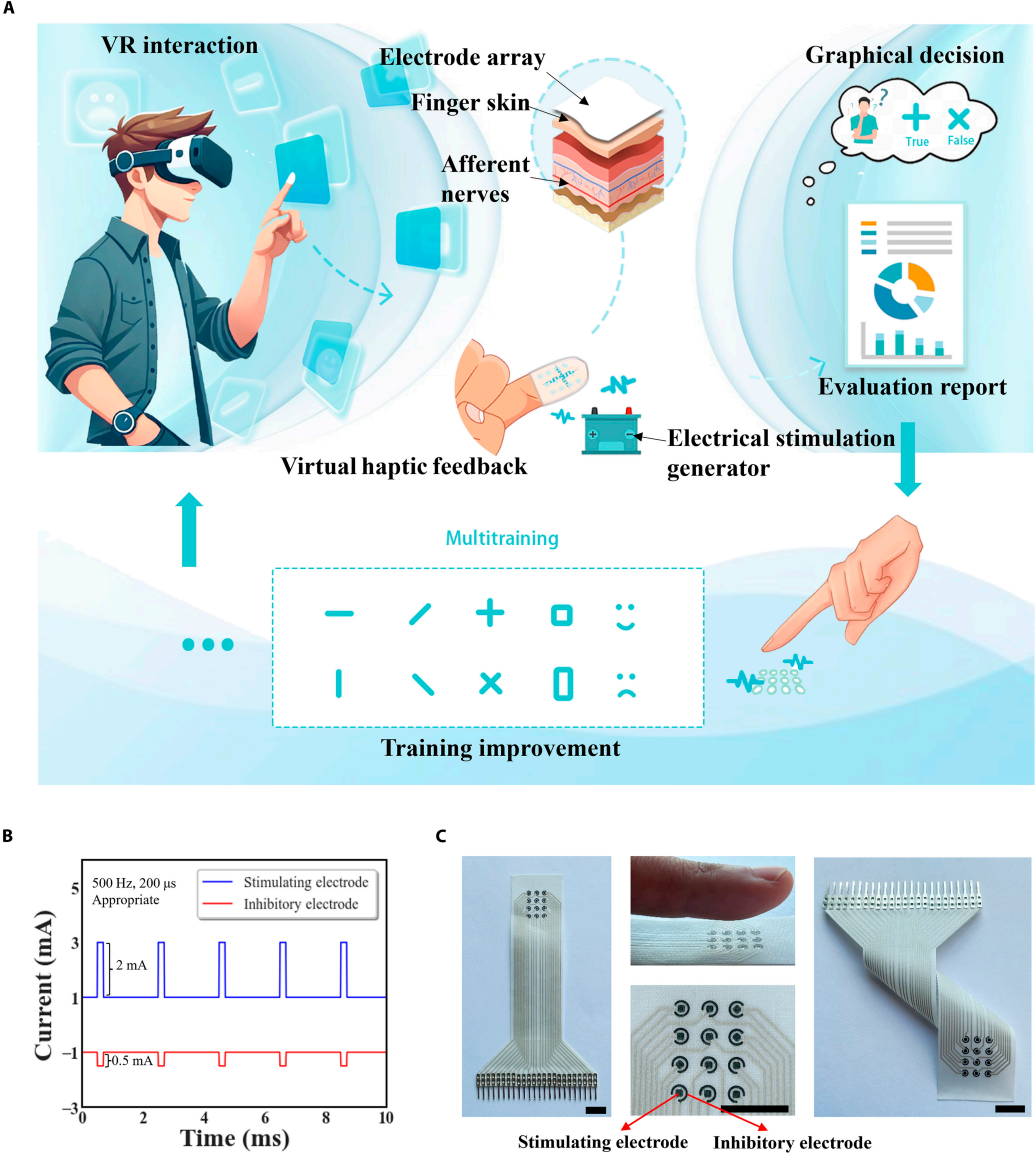
Overview of the tactile perception evaluation interaction system (TPEIS) and its components. (A) Schematic diagram of the TPEIS application in virtual reality (VR). (B) Typical pulsed current waveform for inducing tactile sensation. (C) Front and side optical picture views of the fabric-based microelectrode array (FMA). Scale bars, 1 cm.

The TPEIS consists of an FMA, an electrical stimulation generator, VR glasses, and a VR scene with embedded evaluation rules. The FMA, which is worn by the user on the index finger, simulates multiple tactile patterns through microcurrents supplied by the electrical stimulation generator, and the VR glasses serve as a bridge for visual communication. As shown in Fig. 1A, in the virtual scene, the user clicks on a virtual box (labeled as “+”) with occluded information. The TPEIS stimulates the tactile receptors on the fingertip through the electrode array to form a tactile sensation corresponding to the virtual pattern. At this point, the user needs to identify the shape of the pattern perceived in the tactile feedback of the fingertip and the system will record the result of his judgment and reaction time. After several tests, the system generates an evaluation report to quantify the user’s tactile perception ability. If the user’s tactile perception ability is below the standard deviation (SD) level, the system can also provide repetitive tactile training to gradually improve the user’s tactile sensitivity in the immersive virtual environment.

The electrical stimulation generator (Fig. S1) generates a pulsed square-wave current for the pathway connected to each electrode, where the stimulating electrode is connected to the positive square-wave current, and the inhibitory electrode is connected to the negative square-wave current. The system was set up with a pulse frequency of 500 Hz, a pulse width of 200 μs, and a stimulation current amplitude of 2 mA, where the inhibitory current had an amplitude of one-fourth of the stimulation current, as shown in Fig. 1B. This current parameter provides a comfortable tactile perception experience for most users, as confirmed by the relevant literature and threshold test experiments [9,30–32]. To avoid timing-related confounds, the frequency (500 Hz) and pulse width (200 μs) were fixed across conditions; only the stimulation–inhibition geometry varied. In the area of tactile feedback at the fingertips, the FMA acts as an anode, while the hydrogel patch electrode on the back of the finger acts as a common cathode (Fig. S2). The design allows current to flow through the nerves on the skin side of the fingertip for effective tactile feedback. Since the density of mechanoreceptors on the dorsal side of the finger is much lower than that on the ventral side of the finger, the dorsal electrodes do not elicit tactile stimuli, which prevents interference. Fig. 1C shows the front and side views of the device, with a scale bar of 1 cm. The device substrate comprises a nylon-based textile matrix, which facilitates optimal mechanical compliance and enhanced ergonomic properties. This architectural configuration facilitates optimal tactile feedback delivery while allowing natural finger movements, thereby promoting comfortable use during extended sessions and supporting user comfort.

### Design of the FMA

**Fig. 2.**
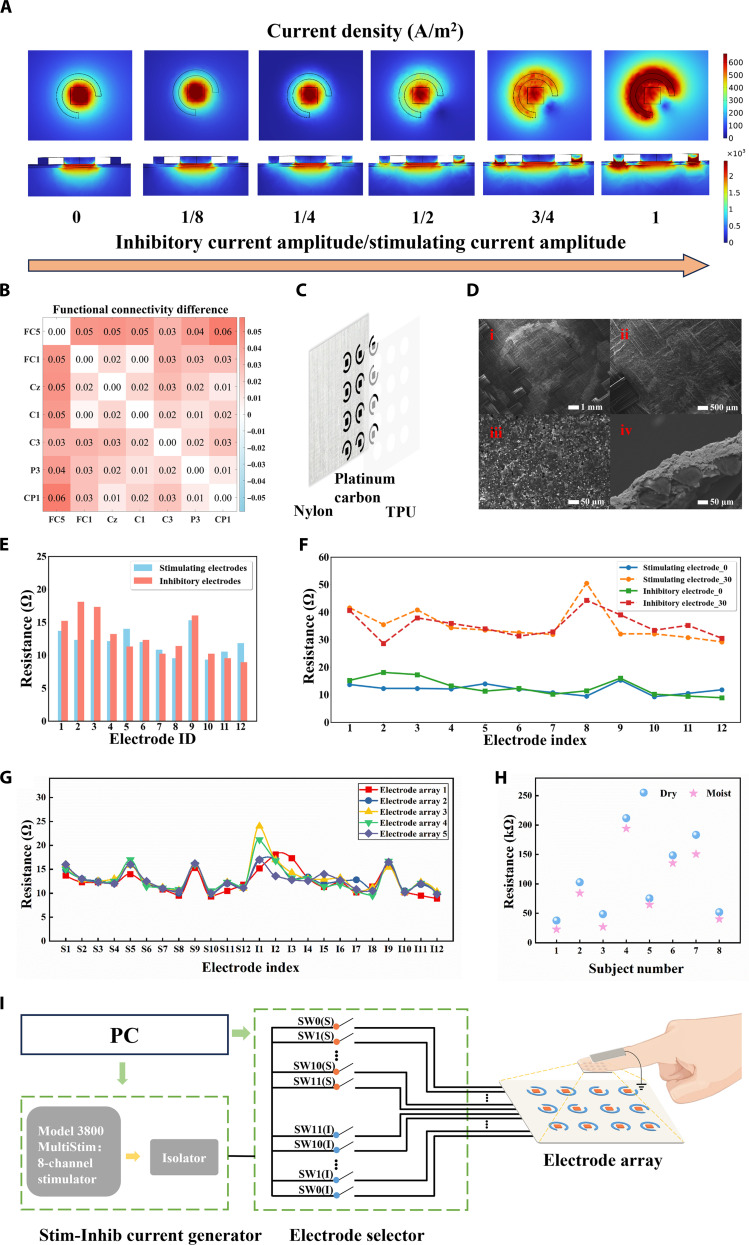
Device design and performance evaluation. (A) Front and cross-sectional simulation result plots of current density distribution under different ratios of current applied to the inhibitory electrode and the stimulating electrode. (B) Group-averaged beta-band functional connectivity difference matrix (with inhibitory electrodes vs without inhibitory electrodes). FC5, FC1, Cz, C1, C3, P3, and CP1 denote EEG electrode positions. (C) Schematic diagram of the device structure. TPU, thermoplastic polyurethane. (D) Scanning electron microscope (SEM) diagram of the device. (E) Resistance result plots for each line in the electrode array. (F) Measured resistance of electrode traces after 30 participants, indicating durability. (G) Line resistance of stimulation and inhibition traces measured from 5 independently fabricated electrode arrays. (H) Electrode–skin contact impedance under dry and moist fingertip conditions. (I) Schematic diagram of the electrical stimulation tactile feedback control system. PC, personal computer.

The precise differentiation of tactile perceptual capabilities is confounded by current diffusion phenomena, which not only compromises perceptual acuity but may also induce varying degrees of paresthesia. To mitigate these limitations, we engineered a novel microelectrode unit wherein the central electrode functions as the stimulation locus while the peripheral electrode serves as an inhibitory element (Fig. S3). The inhibitory electrode employs a counterdirectional waveform relative to the stimulating electrode, with a substantially attenuated current amplitude compared to the stimulation source. This architectural configuration not only constrains stimulation current diffusion and minimizes interference patterns but also facilitates charge neutralization and attenuates the onset of tactile desensitization phenomena. In addition, since the fabric substrate does not facilitate backside wiring, stimulation current introduction requires the inhibitory electrode to open the gap. The effect of this interleaved notched design on the current density distribution was verified by COMSOL simulations, which showed that the adjustment did not affect the effective distribution of the current (Fig. S4). To optimize the amplitude ratio of inhibition to stimulation current, we analyzed the variation of the inhibitory to stimulation current ratio from 0 to 1 by COMSOL simulations. As demonstrated in Fig. 2A, maximal current density localization in the central electrode region was achieved when the inhibitory current amplitude maintained a 1:4 ratio relative to the stimulation current, thereby establishing this as the optimal parametric configuration. A more detailed explanation of the inhibitory field mechanism and its simulation-based validation is provided in Note S1.

Furthermore, to optimize the electrode array architecture, we systematically investigated the parametric influence of central electrode geometry, inhibitory electrode multiplicity, and dimensional characteristics on current density distribution. In electrical stimulation applications, microelectrode geometries predominantly encompass circular and rectangular configurations. By simulation comparison (as shown in Fig. S5), square electrodes perform better in terms of current concentration. Drawing upon established research regarding tactile receptive field dimensions (about 1 to 1.5 cm^2^) and the 2-point tactile threshold (2 to 4 mm) in the normal population, we chose a 3 × 4 twelve-point electrode array layout [3,33,34]. The detailed optimization process of the electrode size and spacing is detailed in Fig. S6.

To provide an additional objective physiological readout in human participants, complementing the simulation-based field analysis, we conducted an exploratory low-density electroencephalography (EEG) study during electrotactile stimulation with versus without inhibitory electrodes (see Note S3 for the experimental paradigm, recording/preprocessing, and connectivity computation; Fig. S7 for traces and power spectra; and Fig. S8 for condition-specific matrices). Beta-band (13 to 30 Hz) functional connectivity was quantified using amplitude–envelope correlation, i.e., Pearson correlations between Hilbert-derived beta amplitude envelopes across electrode pairs. Fig. 2B shows the group-averaged difference matrix (inhibitory − noninhibitory): Most entries are positive (approximately 0.01 to 0.06), indicating a small but broadly distributed increase in beta-band coupling among these sensorimotor/parietal regions of interest when inhibitory electrodes are active. We interpret this finding as preliminary and descriptive while noting that its direction is consistent with the idea that inhibitory electrodes may modulate tactile-related cortical network states in a manner compatible with improved spatial acuity [35–37].

Fig. 2C shows the schematic structure of the FMA device. The device consists of a nylon substrate, platinum–carbon metal electrode, and thermoplastic polyurethane (TPU) insulating film. The nylon substrate is thin, soft, and comfortable to wear; the platinum–carbon metal electrode provides excellent electrical conductivity and abrasion resistance; and the TPU insulating film is low-cost and highly durable for insulating protection and encapsulation of electrode areas. Fig. 2D(i) to (iii) shows scanning electron microscope images of the front side of the device at 40×, 100×, and 1,000× magnifications to demonstrate the details of the platinum–carbon metal electrodes and the surface of the nylon substrate. Fig. 2D(iv) shows a scanning electron microscope view of the cross-section of the device at 1,000× magnification. This cross-sectional view clearly reveals the tight bonding between the platinum–carbon and the nylon substrate. This structural property is key to achieving precise microcurrent manipulation as it helps maintain stable conductivity performance and supports reliable performance under repeated use. For this reason, we choose the platinum–carbon metal electrodes with excellent abrasion resistance. Fig. 2E presents the measured resistance of each conductive trace, showing values below 20 Ω, indicating good baseline conductivity across all channels. Fig. 2F shows that after experiments with 30 participants, the resistance values only slightly increased due to surface wear and sweat exposure, remaining around or below 50 Ω (maximum value of approximately 50.5 Ω). This small change over extensive use demonstrates that the device maintains reliable conductivity and durability, continuing to operate well within safe and functional limits. Notably, the *x*-axis in both figures corresponds to the physical electrode positions numbered from 1 to 12 (as illustrated in Fig. S9), rather than arbitrary indices, ensuring that each resistance value is mapped to its actual spatial location on the array. Furthermore, Fig. 2G shows that line-resistance profiles measured from 5 independently fabricated arrays are nearly overlapping across all 24 stimulation and inhibition traces, indicating high device-to-device uniformity in the electrode routing (Note S4). Fig. 2H further shows that the electrode–skin impedance measured under dry and naturally moist fingertip conditions decreases with moderate sweating while remaining within the safe operating range of the constant-current stimulator (Note S5). These results suggest that typical variations in fingertip moisture do not compromise stimulation reliability and may even make electrotactile sensations slightly easier to perceive.

To achieve effective feedback control of electrical stimulation, we constructed a comprehensive control system, as illustrated in Fig. 2I. The system’s operational protocol encompasses a hierarchical signal transduction cascade: The computational interface transmits control parameters to the Stim-Inhib current generator, which generates precisely modulated stimulation currents. These currents are subsequently propagated through the electrode array matrix to facilitate haptic feedback, with the circuit completion achieved via a dorsal digital ground electrode. Temporal sequencing protocols govern the generation of microcurrent-based tactile patterns, while a multichannel selector module enables sequential control implementation (as illustrated in Fig. S10). This system not only enhances the flexibility of control but also improves the accuracy and reproducibility of the experiments by detailing the design.

### Fabrication of the FMA

Nylon was selected as the fabric substrate, and a high-temperature thermo-compositing process was used to combine a TPU insulating film with the nylon substrate, resulting in a composite material with flexibility and wear resistance. The electrode material used was carbon platinum (FZ-BT9505, manufactured by Guangdong Fangzhou Manufacturing Technology Co., Ltd.), consisting of nano-pure carbon powder, carbon nanotubes, and platinum powder. The nano-pure carbon powder provides basic conductivity, the carbon nanotubes enhance the mechanical strength and flexibility of the electrodes, and the platinum powder improves their chemical stability and corrosion resistance. The conductor material is silver paste (FZ-DJ086, Fangzhou brand), which contains flake silver powder, spherical silver powder, and silver chloride powder. The flake silver powder improves conductivity, the spherical silver powder enhances fluidity and printing uniformity, and the silver chloride powder increases material stability. Both metal materials are combined with polyvinylpyrrolidone as a binder and polyvinyl alcohol to improve mechanical strength, while epoxy resin is used to enhance wear resistance and durability. To ensure the uniform distribution of the silver paste, dibasic ester solvent was used to adjust its flowability. The mixed materials were processed by high-speed stirring to ensure good conductivity and uniformity of the carbon platinum and silver paste. Finally, the designed electrode array pattern was deposited onto the composite substrate using a screen-printing process. After printing, the substrate undergoes one round of baking: one at 150 °C for high-temperature curing to remove solvents. In the end, a TPU insulating film is coated around the electrode layer to ensure electrical insulation of the non-electrode areas and the safety of the device.

### Control system

Our control system integrates Unity for tactile interaction within virtual environments. When a collision between the virtual hand and virtual objects is detected by Unity’s collision algorithm, commands are sent to the Model 3800 electrostimulator and the multichannel switching circuit. The Model 3800 electrostimulator generates electrical stimulation, while the switching circuit determines which tactile feedback pattern will be delivered to the user. The stimulation is delivered as unipolar pulses, with parameters such as amplitude, pulse width, and frequency adjustable through the software interface. The multichannel switching system, controlled by a microcontroller (STM32), manages the sequential activation of the electrode array through 74HC595 shift registers and ULN2803 Darlington arrays. The system’s modular design allows for expanding the number of channels by cascading multiple 74HC595 modules. The switch matrix independently controls each electrode, ensuring that the required timing between stimulating and inhibitory electrodes is maintained.

The driving capability of the electrode array is enhanced by the ULN2803 array, ensuring precise control of each electrode via solid-state relays. The system is designed for scalability, high reliability, and consistent performance, providing robust hardware support for delivering precise microcurrent tactile feedback within virtual environments. The modular design ensures stable operation and reliable signal transmission, which is essential for providing seamless virtual tactile experiences.

## Results

### Verification of the inhibitory electrode effect and data acquisition

**Fig. 3.**
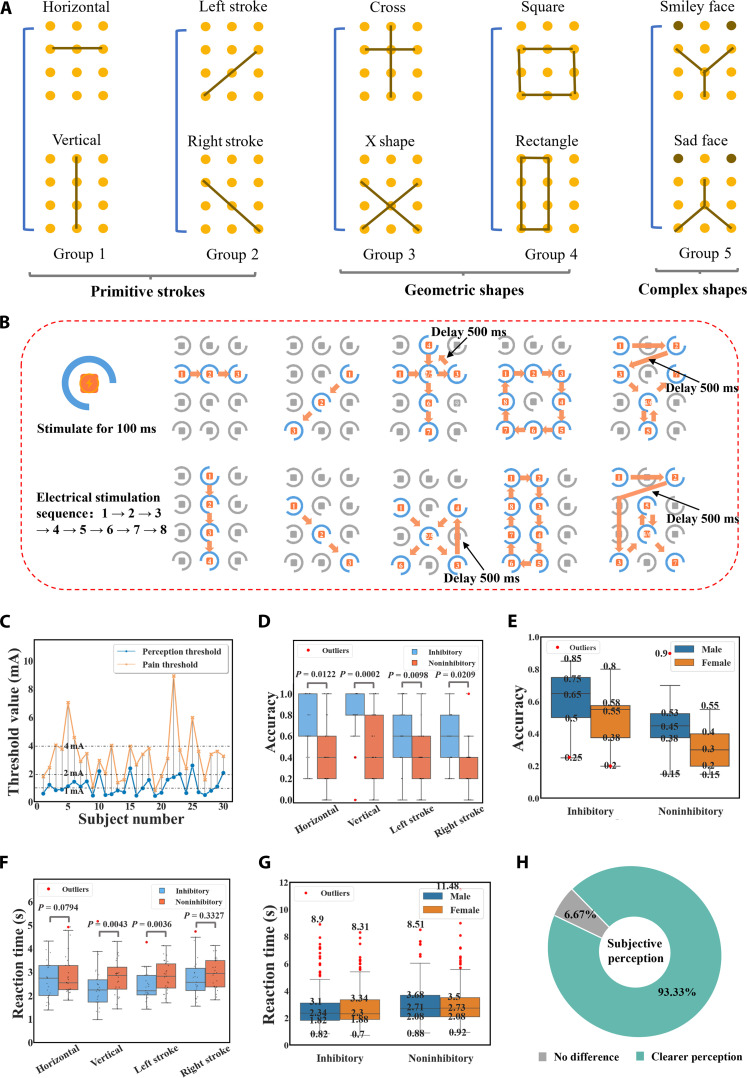
Comparison of tactile perception with and without inhibitory electrodes in 30 participants. (A) Five groups of virtual tactile simulation patterns, categorized by complexity into primitive strokes, geometric shapes, and complex shapes, to support progressive tactile perception assessment. (B) Sequence of 10 patterns of electrical stimulation modality. (C) Perception thresholds and pain thresholds of the 30 participants. (D) Comparison of recognition accuracy of primitive stroke patterns (horizontal, vertical, left stroke, and right stroke) among the 30 participants under conditions with and without inhibitory electrodes. (E) Comparison of recognition accuracy between male and female participants under conditions with and without inhibitory electrodes. (F) Comparison of reaction times for primitive stroke patterns among the 30 participants under conditions with and without inhibitory electrodes. (G) Comparison of reaction times between male and female participants under conditions with and without inhibitory electrodes. (H) The 30 participants were assessed for their feelings about whether or not inhibitory electrodes were used, with a pie chart showing the proportion of those who felt a noticeable difference (feeling clearer) versus those who felt no noticeable difference.

For the assessment of human tactile perception ability, we designed a microcurrent simulation experiment with 5 sets of patterns, as shown in Fig. 3A. These patterns were divided into 3 main categories, namely, primitive strokes (horizontal, vertical, left stroke, and right stroke), geometric shapes (cross, X shape, square, and rectangle), and complex shapes (smiley face and sad face). Primitive strokes test the perception of direction and length. Geometric shapes introduce elements of shape and spatial relationships. Complex shapes involve multiple visual features such as points, lines, angles, and symmetry. This combination of patterns was chosen with the aim of striking a balance between experimental complexity and feasibility, covering a wide range of tactile perceptual dimensions without unduly increasing the complexity of the experimental design.

Fig. 3B illustrates the spatiotemporal characteristics of the electrical stimulation paradigm across diverse patterns. The sequential activation protocol within the electrode array matrix follows a prescribed numerical progression (1 to 8) as indicated by directional vectors. Each electrode locus receives a 100-ms stimulation duration, with interstimulus intervals standardized at 1 ms, except where explicitly designated as 500 ms. To validate the effectiveness of inhibitory electrodes and quantitatively assess tactile perception capabilities, we conducted 2 sets of experiments, collecting 2 separate datasets. The first set focused on evaluating the effect of inhibitory electrodes on tactile perception. The second set was designed to establish the tactile perception evaluation system and assess participants’ ability to recognize various tactile patterns. We recruited 30 participants between the ages of 18 and 28 with balanced gender distribution. All of the volunteers gave written informed consent about the experimental procedure. The experiments with human subjects were performed in compliance with all the ethical regulations under a protocol that was approved by Zhujiang Hospital of Southern Medical University. All participants were trained to manipulate the electrotactile system with the help of experimenters until they understood the sensation of electrical stimulation. Detailed experimental data collection protocols for both experimental paradigms are provided in Note S2.

To analyze the effectiveness of inhibitory electrodes, we begin with the data collected from the first set of experiments. Due to individual differences in perception thresholds, a threshold test was conducted prior to the start of the experiment. Fig. 3C shows the perception and pain thresholds of 30 participants with inhibitory electrodes, with the median value of these thresholds used as the operational threshold during the experiments. The experimental data demonstrated that operational threshold amplitudes predominantly clustered around 2 mA, with outlying values distributed between 1 and 4 mA across the participant population. Based on this finding, the system was set to have 3 current levels, 1, 2, and 4 mA, to accommodate different sensory thresholds. As shown in Fig. 3D, the box plots compare recognition accuracy for primitive stroke patterns (horizontal, vertical, left stroke, and right stroke) under both inhibitory and noninhibitory electrode conditions. The distributions reveal a consistently higher median accuracy with inhibitory electrodes, accompanied by narrower interquartile ranges and fewer extreme outliers, suggesting a more stable and reliable recognition performance. After confirming the approximate normality of the within-subject difference scores with Shapiro–Wilk tests (all *P* > 0.05, *n* = 30), we conducted paired-samples *t* tests (2-tailed, *α* = 0.05). Accuracy improvements were significant for all 4 patterns (*P* < 0.05), with particularly strong effects for the vertical (*P* = 0.0002) and left-stroke (*P* = 0.0098) patterns. Although the group-level effect was robust, 3 participants exhibited inverse response patterns, which may reflect individual variability in somatosensory processing or methodological factors. Fig. 3E further illustrates the distribution of recognition accuracy across male and female participants. While both groups benefit from the use of inhibitory electrodes, male participants show a slightly higher median accuracy and less variability compared to their female counterparts. These differences are descriptive and secondary to the primary within-subject effect of inhibition. Reaction times are summarized in Fig. 3F and G. The box plots indicate a general trend of shorter median response times with inhibitory electrodes, along with reduced variability across most stroke patterns. This acceleration reached statistical significance for the vertical and left-stroke patterns (*P* = 0.0043 and *P* = 0.0036, respectively), whereas no significant differences were observed for the horizontal (*P* = 0.0794) and right-stroke patterns (*P* = 0.3327). These findings suggest that inhibitory electrodes not only enhance recognition accuracy but also facilitate faster and more consistent tactile responses, although the degree of improvement varies with stroke orientation. Fig. 3H displays a pie chart of the subjective perceptions of the 30 participants regarding the presence or absence of inhibitory electrodes. In the feedback, 93.3% of participants felt that tactile perception was clearer and more comfortable when using inhibitory electrodes. Additionally, Fig. S11 explores correlations between perception thresholds, pain thresholds, body mass index, and gender. The correlation matrix indicates no significant associations among these variables. Fig. S12 presents a comparative analysis of threshold differentials between conditions with and without inhibitory electrodes, revealing consistently reduced threshold values in the presence of inhibitory electrodes. These findings substantiate the efficacy of inhibitory electrode configurations in augmenting tactile stimulus precision while attenuating nontarget stimulation interference.

### Establishment of the tactile perception assessment system

**Fig. 4.**
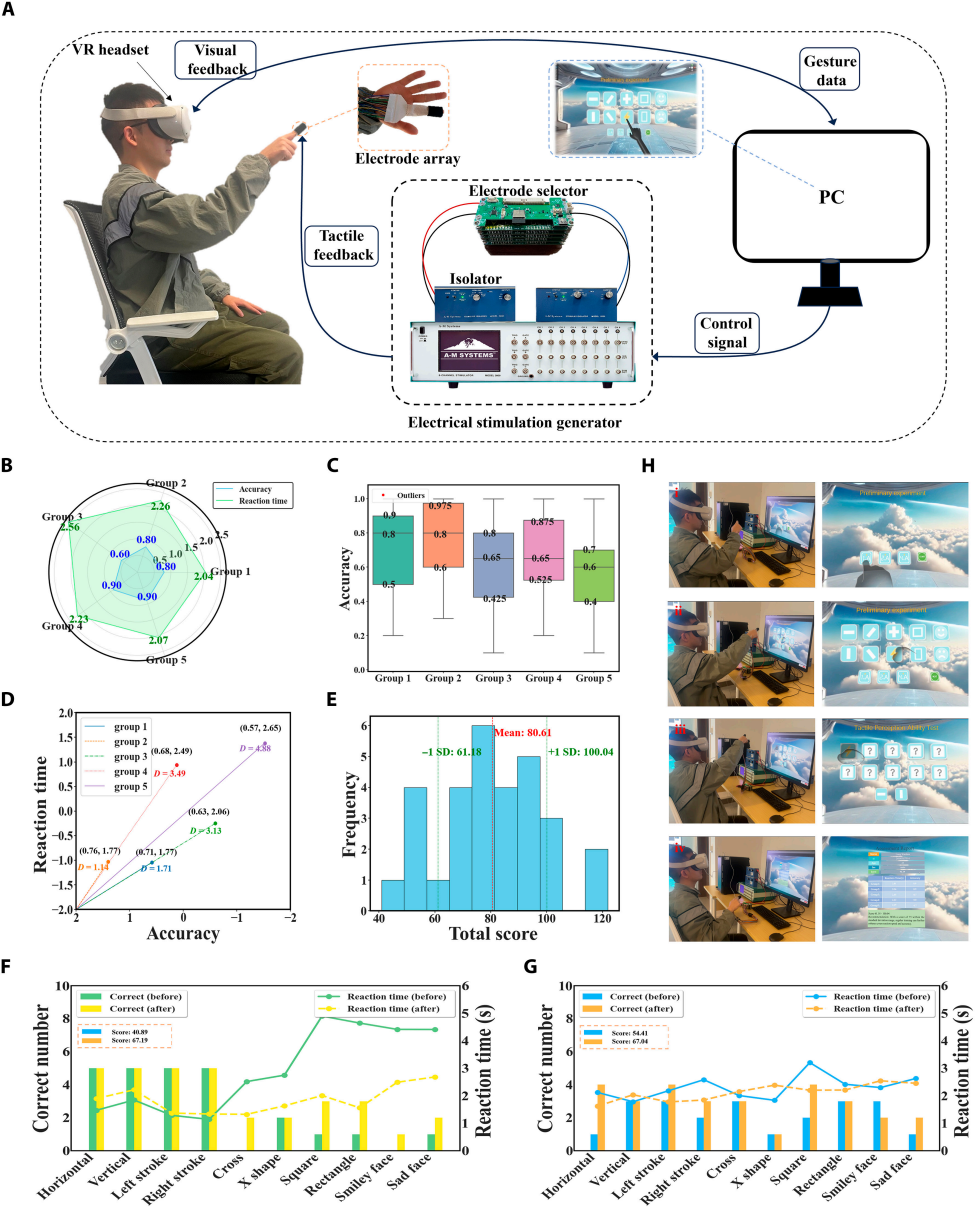
Data-driven tactile pattern recognition and evaluation methodology. (A) Illustration of a user wearing the TPEIS. A VR camera tracks their hand movements and sends data to a computer, which commands the tactile feedback module (electrostimulation generator) to deliver tactile stimuli to the fingers upon touching different virtual patterns. (B) Radar chart of accuracy and reaction time data for participant ID 4. (C) Box plots of average recognition accuracy for 5 groups of tactile patterns among 30 participants. (D) Quantification of pattern difficulty levels based on normalized Euclidean distance, where group 2 (with the shortest distance) is used as the reference group with a score of 1. (E) Distribution of standard deviations for tactile cognition scores among 30 participants. (F and G) Changes in tactile perception scores for participant IDs 8 and 9 before and after training. (H) Demonstration of a user operating the TPEIS. The diagram illustrates 4 key stages: (i) selecting an appropriate stimulation threshold, (ii) experiencing known tactile patterns for familiarization, (iii) identifying unknown patterns during formal testing, and (iv) generating an automated evaluation report with individualized scores and training suggestions.

Based on the second set of data collected to establish the tactile perception evaluation system, Fig. 4A illustrates the use of tactile interfaces in immersive VR applications for interacting with the virtual world. The user wears VR goggles, with a soft tactile feedback electrode array on their fingers. Commercial VR head-mounted displays (HMDs) provide visual information as well as hand motion capture. Tactile feedback, synchronized with the HMD, generates tactile sensations from the video content, which is created using the Unity3D game engine. The motion controllers of the HMD synchronize with the engine to track the user’s hand movements, detect collisions between the virtual hand and virtual objects, and send commands to the tactile interface to obtain the corresponding tactile feedback.

The assessment of tactile perception ability depends on both the accuracy of pattern recognition and factors such as the subject’s reaction time and the difficulty level of the patterns. Fig. 4B shows the radar chart of accuracy and reaction time for subject no. 4 during their personal trials. Fig. 4C and Fig. S13 present the average accuracy and reaction time for 30 participants across 5 pattern groups, visualized as box plots to highlight the distribution and central tendency within each group. These 5 groups are organized into 3 categories of tactile complexity: group 1 and group 2 represent primitive strokes patterns, group 3 and group 4 contain geometric shapes, and group 5 includes complex patterns. While the 5-group structure serves experimental organization and scoring purposes, the 3-category classification reflects the intended perceptual difficulty. As the patterns transition from primitive strokes to geometric forms and then to complex emotional shapes, the results show a general decline in recognition accuracy and an increase in reaction time, indicating a progressive rise in task difficulty.

To evaluate subjects’ tactile discrimination capabilities while accounting for pattern complexity, we employed Euclidean distance metrics as a quantitative measure of pattern differentiation across groups. This methodological approach enables the systematic assessment of pattern difficulty by measuring the geometric dissimilarity between stimulus pairs, where larger distances indicate increased perceptual challenges and smaller distances suggest more readily distinguishable patterns. The variables involved in this method include the accuracy and reaction time of the 30 subjects. Since the data ranges of these 2 variables are different, we first standardized the data. Accuracy (*A*) and reaction time (*R*) are defined as the 2 key variables for pattern recognition, and both variables are standardized first.

$$A_{\mathrm{std}}=\frac{A-\mu_{A}}{\sigma_{A}}$$ (1)

$$R_{\mathrm{std}}=\frac{R-\mu_{R}}{\sigma_{R}}$$ (2)

where $\mu_{A}$ and $\mu_{R}$ are the means of accuracy and reaction time, respectively, and $\sigma_{A}$ and $\sigma_{R}$ are the corresponding SDs. Then, as shown in Fig. 4D, the overall average accuracy and reaction time of each pattern group are used as the coordinates (with the *x*-axis range set to [2, −2] and the *y*-axis range set to [−2, 2]), and the origin is set at (2, −2). Proximity to the coordinate origin correlates positively with recognition accuracy and inversely with response latency, indicating the enhanced perceptual accessibility of these patterns during tactile discrimination tasks. Next, the Euclidean distance *D* between the standardized accuracy and reaction time for each pattern group is calculated to assess its difficulty level.

$$D=\sqrt{{\left(A_{\mathrm{std}}-2\right)}^{2}+{\left(R_{\mathrm{std}}+2\right)}^{2}}$$ (3)

Among all groups, group 2 demonstrated the shortest Euclidean distance, indicating the highest recognizability (i.e., highest accuracy and fastest response). Therefore, we selected group 2 as the reference group, assigning it a difficulty score of 1 point. The difficulty scores of other groups were then calculated relative to group 2 as follows:

$$\text{Difficulty score of group}\;i=\frac{D_{i}}{D_{\text{reference}}}$$ (4)

where $D_{\text{reference}}$ is the Euclidean distance for the reference group. The calculated scores indicate the difficulty levels for each pattern group, with scores as follows: “group 1”, 1.5; “group 2”, 1.0; “group 3”, 2.75; “group 4”, 3.06; and “group 5”, 4.28. This framework for quantifying based on difficulty levels allows us to determine the tactile perception scores of the 30 subjects, including both average and SD. As depicted in Fig. 4E, the score distribution is shown in histogram format, with the *x*-axis representing the score range and the *y*-axis showing the number of subjects within each score range. A red dashed line indicates the average score of all subjects at 80.61 points. This suggests that the performance of most subjects is near the average, with scores primarily concentrated in this area. A green dashed line representing the SD reflects the spread of scores. The SD positions are +1 SD at 100.04 points and −1 SD at 61.18 points, indicating that about 68% of the subjects’ scores fall within this range. The distribution of performance metrics demonstrates homogeneity across participants, with scores exhibiting central tendency. Participants performing below the threshold of 61.18 may exhibit reduced tactile sensitivity, suggesting potential benefit from remedial intervention, while those exceeding 100.04 points demonstrate exceptional tactile discrimination capabilities. In a subset of 8 participants, we further measured mechanical detection thresholds at the right index fingertip using a Semmes–Weinstein monofilament kit (Aesthesio, USA) [38]. The experimental paradigm is described in Note S6, and the device is shown in Fig. S14. The resulting monofilament thresholds were negatively correlated with the corresponding TPEIS scores (Pearson *r* = −0.81 and *P* = 0.016; Fig. S15), providing preliminary convergent validity between the TPEIS metric and a widely used clinical tactile threshold test.

After evaluating the system, we found that some participants had tactile perception scores below the SD threshold. Five such low-performing participants (baseline scores < −1 SD, i.e., <61.18 points) were invited for a follow-up session. They first received a single 15-min pattern-based electrotactile practice block in the VR environment and then completed 3 consecutive TPEIS tests under identical task conditions without additional training between tests. As summarized in Fig. S16, all 5 participants showed higher scores posttraining than at baseline, and the 3 posttraining tests for each individual clustered within a relatively narrow range, indicating both a short-term training effect and reasonable test–retest reliability of the TPEIS score. Fig. 4F and G illustrates the improvement in the tactile perception performance of 2 representative participants (IDs 8 and 9) following 15 min of training. The left *y*-axis indicates the number of correctly recognized patterns (maximum of 5 per pattern), while the right *y*-axis shows the corresponding reaction time (in seconds). Bar charts compare recognition accuracy before and after training, and line plots display reaction time trends. For subject 8, the perception score improved from 40.89 to 67.19, while for subject 9, the score increased from 54.41 to 67.04, indicating that brief training can enhance both recognition accuracy and response efficiency.

We have developed a Unity-based TPEIS for quantitatively assessing tactile perception abilities in a virtual environment (Movie S1). This system is set in a virtual space station environment, where subjects can perceive virtual touch by touching various patterned information boxes. This methodological approach enhances participant engagement while substantially increasing ecological validity and experimental immersion. TPEIS effectively avoids the spatial constraints and adverse factors faced during tactile perception assessments in real environments. As shown in Fig. 4H(i), the threshold selection interface allows subjects to choose the appropriate microcurrent levels (1, 2, or 4 mA) before the experiment. The experimental protocol requires participants to wear a VR headset and manipulate interface controls via virtual hand interactions to experience varying current intensities, thereby establishing optimal threshold parameters for subsequent experimental conditions. Fig. 4H(ii) presents the pre-experimental process before the experiment starts, where subjects familiarize themselves with the feeling of tactile perception by experiencing microcurrent stimulation from different patterns. In this phase, virtual fingers generated by Ultraleap 3Di technology in the VR goggles touch the patterns on the screen, which turn into a lightning symbol to indicate the start of microcurrent stimulation. The electrical stimulator generates microcurrents based on the control system and stimulates the subjects through tactile feedback. Fig. 4H(iii) illustrates the process of testing and interacting with 5 groups of patterns. In this section, subjects click on unknown question mark patterns in the virtual boxes and judge the corresponding pattern types through tactile perception. The system records each judgment result and reaction time to further quantify tactile perception abilities. Fig. 4H(iv) is the tactile perception evaluation report, which is generated based on the subjects’ judgment results and experimental rules. The report includes tactile perception scores and provides recommendations based on the scores. Scores falling below 1 SD threshold prompt system-generated recommendations for perceptual enhancement through structured electrical stimulation training protocols.

**Fig. 5.**
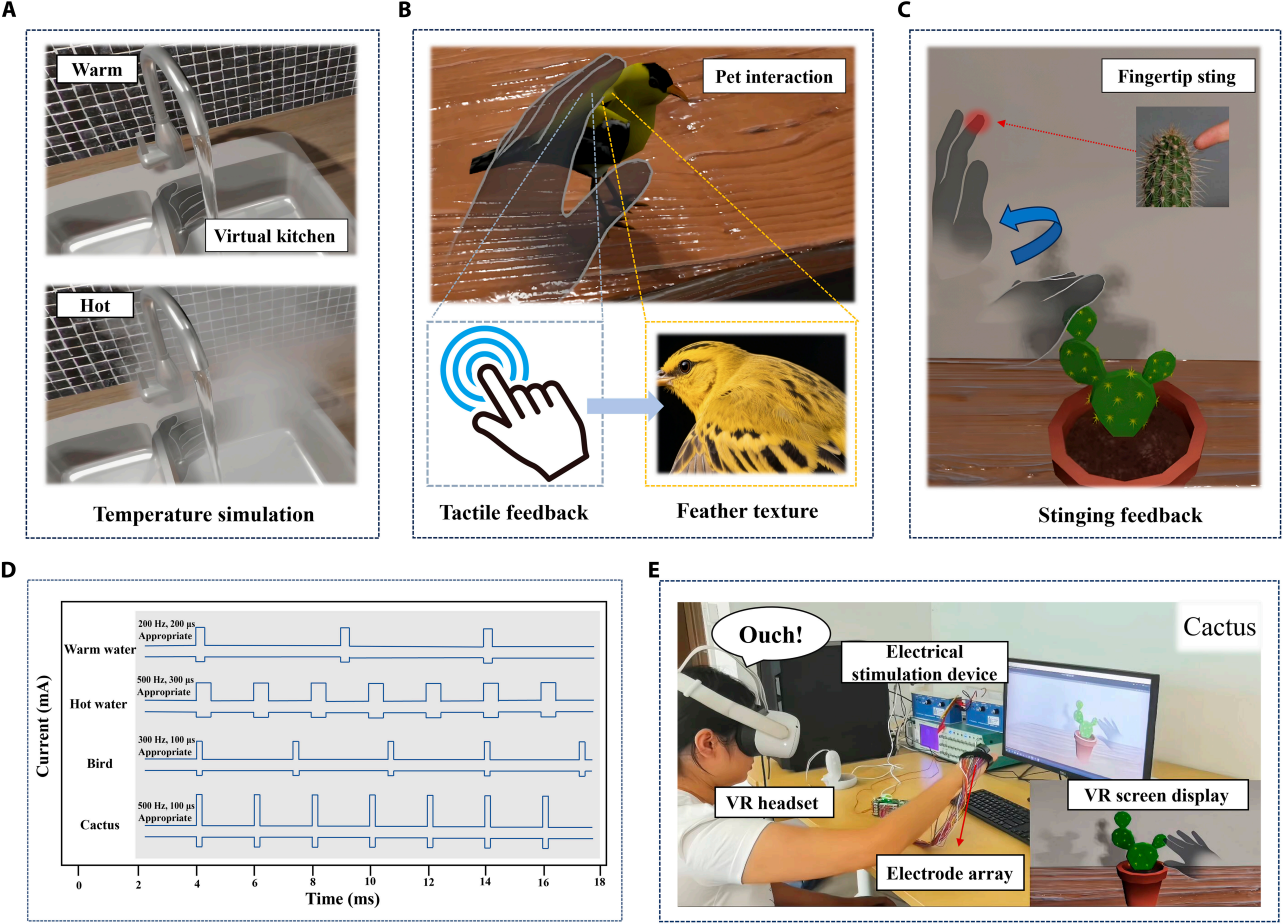
Demonstration of immersive VR scenarios enabled by the electrotactile system. (A) Virtual kitchen environment simulating the tactile experience of warm and hot running water through tailored stimulation parameters. (B) Pet interaction scenario, where users experience fine tactile feedback while stroking a bird’s forehead. (C) Cactus interaction scenario, evoking a sharp, stinging tactile sensation when the fingertip contacts cactus spines. (D) Corresponding stimulation waveforms for each scenario, illustrating variations in frequency, amplitude, and pulse width to achieve distinct tactile effects. (E) Experimental setup showing the VR headset, electrode array, and stimulation device used during the cactus interaction task.

In addition to tactile assessment, this electrotactile stimulation system demonstrates significant advantages within immersive VR experiences, particularly in enhancing tactile clarity and comfort. Benefiting from an innovative electrode design, the system effectively suppresses current diffusion while enhancing focusing capabilities, ensuring precise and comfortable tactile feedback. In the virtual kitchen environment (Fig. 5A), users can perceive the warmth and heat of the water flow through sequential activation of electrode arrays and precisely controlled stimulation parameters, enhancing the realism of the interaction (Movie S2). The pet interaction scenario (Fig. 5B) allows users to experience delicate tactile feedback when stroking a bird’s forehead (Movie S3). The cactus touch scenario (Fig. 5C) employs single-point stimulation to simulate the sharp pain of contact with cactus spines, maintaining the intensity of the stimulation while preventing numbness in users (Movie S4). Detailed electrode activation sequences and stimulation settings are provided in Fig. S17.

These scenarios are intended not only to demonstrate the effectiveness of tactile feedback but also to deeply validate the system’s application in complex interactive tasks. By simulating various daily tactile experience scenarios, such as touching water, interacting with animals, and contacting sharp objects, the electrotactile system’s ability to transmit diverse tactile information is showcased, further enhancing the realism and immersion of the virtual environment. Fig. 5D shows the corresponding stimulation waveforms for each scenario, with careful adjustments of frequency, amplitude, and pulse width to ensure the desired tactile effects for each scene. Fig. 5E presents the experimental setup, where users wear a VR headset, and the electrode array and stimulation device work in harmony to create these engaging immersive experiences.

## Discussion

In summary, this study presents an innovative fabric-based electrical stimulation tactile perception enhancement system (TPEIS). By integrating microcurrent stimulation with VR technology, we were able to quantitatively assess tactile perception abilities in an immersive and interactive environment. Central to the system’s performance are the electrode arrays incorporating a stimulation–inhibition electrode unit structure, specifically designed to improve tactile feedback precision by effectively mitigating current diffusion between adjacent electrodes. The fabric-based design ensured flexibility and comfort and was designed to support extended-session use without compromising signal stability. Through microcurrent stimulation simulations and quantitative experiments, we demonstrated that the inhibitory electrodes substantially enhanced tactile perception accuracy and reduced interference from nontarget stimuli. Unlike concentric-ring electrodes used to suppress far-field stimulation artifacts in bidirectional HMIs (e.g., Yang et al. [29]), our stimulation–inhibition unit acts locally to curb near-field lateral current spread between adjacent pads in dense arrays, thereby sharpening spatial excitation while preserving channel density. Experimental results showed marked improvements in both recognition accuracy and reaction time under conditions involving inhibitory electrodes. This integration of VR and electrotactile feedback not only provides a flexible platform for tactile training and evaluation but also enables immersive and dynamic user experiences, as evidenced by real-world scenarios such as interacting with running water, stroking a bird’s forehead, and feeling a cactus. These applications highlight the potential for diverse future uses in tactile training, neurorehabilitation, and advanced smart wearable devices. In healthy young adults under controlled laboratory settings, these findings demonstrate the feasibility of delivering precise, individualized electrotactile feedback. Translation to broader demographics and clinical cohorts will require additional testing.

Nevertheless, several aspects remain beyond the scope of the current investigation due to inherent methodological and practical constraints. First, the present study deliberately recruited young adult participants (18 to 28 years old) to control for potential confounding factors such as age-related variability in tactile perception. Accordingly, the current results should not be generalized to older adults or sensory-impaired users without further testing. Expanding this research to more heterogeneous populations (e.g., older adults or individuals with sensory impairments) would necessitate additional ethical considerations, a larger recruitment effort, and longer-term follow-up, all of which are planned for subsequent research phases. Second, while the stimulation–inhibition electrode unit configuration demonstrated effectiveness within our controlled laboratory conditions, a comprehensive investigation into skin–electrode interface dynamics—such as variations in skin impedance, hydration, and mechanical properties—would require more specialized, prolonged, or invasive testing protocols. These factors could potentially affect electrotactile stimulation quality and perceptual reliability but were outside the specific validation objectives of the current work. Third, neurophysiological support for the proposed mechanism is preliminary but encouraging: The exploratory low-density EEG analysis in the Supplementary Materials showed a small yet broadly distributed, directionally consistent increase in beta-band functional coupling (amplitude–envelope correlation) when inhibitory electrodes were active. However, given the small sample size, low-density montage, and descriptive nature of this pilot analysis, future studies using larger cohorts and higher-density EEG or magnetoencephalography are needed to confirm and localize configuration-specific changes in somatosensory cortical network dynamics. Fourth, the present protocol was participant blinded (single blind) rather than double blinded: Participants were not informed of the condition and trials were randomized, but the experimenter was not blinded at run time, leaving the possibility of residual expectancy/handling bias. To strengthen internal validity, future studies will adopt a fully double-blind protocol in which condition assignment is computer controlled using coded labels with a masked operator interface, and unblinding occurs only after data lock. Fifth, although electrode impedance and morphology remained stable across repeated uses and brief postsession inspection revealed no visible erythema, the current single-session design did not include a dedicated long-term safety and comfort assessment (e.g., continuous contact-temperature logging or standardized skin-integrity assays). Consistent with prior electrotactile literature that reports irritation-free contact for textile/hydrogel microcurrent interfaces under standard operating ranges and highlights the role of stimulation duration/waveform in perceived comfort [9,11,39], our breathable nylon–TPU substrate and platinum–carbon electrodes likely contributed to the absence of discomfort in this study; however, extended-wear safety must be established prospectively.

Notably, the TPEIS workflow includes per-participant threshold calibration and adjustable current amplitude, which are expected to accommodate inter-individual differences in electrotactile sensitivity across age and skin conditions; however, this assumption remains to be tested. We therefore plan a prospective study enrolling older adults (≥60 years) and sensory-impaired cohorts using the same workflow. We will record recognition accuracy and reaction time at individualized thresholds, along with comfort ratings and any adverse events, to assess usability and safety. In parallel, we will implement an extended-wear, fully double-blind protocol that incorporates continuous contact-temperature monitoring, standardized skin-integrity assessments (erythema scoring and transepidermal water loss), and repeated comfort/adverse-event tracking to rigorously evaluate long-term safety and tolerability. Stratified analyses by age and skin condition will delineate boundary conditions for generalizability.

Future investigations could systematically address these limitations by including broader demographic samples, incorporating detailed biophysical modeling, and expanding the complexity of interactive tasks. Real-time adaptive stimulation strategies, potentially guided by physiological feedback mechanisms (e.g., skin impedance or neural activity patterns), also represent promising directions for enhancing personalized and context-aware haptic experiences. By carefully considering these future directions, subsequent work can progressively advance electrotactile feedback technologies toward greater robustness, scalability, and user-centric design, ensuring that they effectively meet the diverse needs of practical applications.

## Data Availability

All technical details for producing the figures are enclosed in Materials and Methods and Supplementary Materials. Data are available from the corresponding author L.S. upon reasonable request.

## Supplementary Materials

Supplementary 1 Notes S1 to S6 Movies S1 to S4 Figs. S1 to S17 Tables S1 and S2
