# Supplementary material for: Wearable Fabric Electrotactile System with Stimulation–Inhibition Electrode Units
Source: Cyborg Bionic Syst. 2026 Apr 1;7:0515. doi: 10.34133/cbsystems.0515 (PMC13039521; doi:10.34133/cbsystems.0515)
Supplement: Supplementary 1 — Notes S1 to S6 Movies S1 to S4 Figs. S1 to S17 Tables S1 and S2 [file cbsystems.0515.f1.zip › Revised supplementary information-Highlighted.docx]

Supplementary Information for

**Wearable Fabric Electrotactile System with Stimulation–Inhibition Electrode Units**

Hongbo Yao *et al*.

Corresponding authors: Lin Shu: [shul@scut.edu.cn](mailto:shul@scut.edu.cn), Lei Wei: lei.wei@deakin.edu.au , Xinge Yu: [xingeyu@cityu.edu.hk](mailto:xingeyu@cityu.edu.hk), Xiangmin Xu: xmxu@scut.edu.cn

**This PDF file includes:**

Supplementary Notes S1 to S6

Captions for Supplementary Movies 1 to 4

Supplementary Figs. S1 to S17

Supplementary Tables S1 to S2

**Other Supplementary Materials for this manuscript include the following:**

Supplementary Movies S1 to S4

**Note S1. Mechanism of the** **stimulation–inhibition electrode unit.**

To improve the spatial resolution of electrotactile stimulation, we designed a stimulation–inhibition electrode unit structure in which a low-amplitude reverse-polarity current is applied to the peripheral electrodes surrounding the central stimulation site. This approach aims to mitigate the lateral current diffusion typically observed in multi-electrode arrays due to the heterogeneous conductivity and dielectric characteristics of the skin. In conventional configurations, electrical current tends to spread laterally across the skin and subdermal layers, resulting in a dispersed and blurred current density profile. This dispersion may lead to unintended stimulation of adjacent areas and reduce the precision of activating specific mechanoreceptors such as Meissner corpuscles or Merkel cells, thereby degrading tactile localization and recognition performance.

The stimulation–inhibition electrode unit introduces a low-potential electric boundary around the central electrode. By applying a reverse-polarity current (typically one-fourth of the main stimulation amplitude) to the surrounding electrodes, an opposing electric field is generated. This field redistributes the spatial potential landscape, effectively forming an “electric potential fence” that suppresses outward current leakage and enhances vertical penetration toward target receptors. As demonstrated in Figure 2a of the main text, we conducted finite element simulations of current density distributions under varying inhibitory-to-stimulation current ratios (0, 1/8, 1/4, 1/2, 3/4, and 1). When no inhibitory current is applied, current disperses widely, and the high-density region is poorly defined. Introducing a moderate inhibitory current (e.g., 1/8 or 1/4) results in a more focused and intensified current peak directly beneath the stimulating electrode. The 1:4 ratio yields the most compact and intense central focus, minimizing peripheral activation. However, increasing the inhibitory current beyond this optimal point causes the surrounding electrodes to generate sufficient reverse current to themselves activate peripheral mechanoreceptors. This may lead to multi-point perceptual confusion or sensory numbness, counteracting the intended focusing effect. Therefore, we define the 1:4 ratio as a safe and effective balance between spatial precision and minimal off-target stimulation.

This mechanism provides theoretical support for the enhanced performance observed in our pattern recognition experiments under the inhibitory condition. It also confirms that the stimulation–inhibition electrode unit structure design enhances spatial tactile resolution not by increasing current strength, but by controlling current trajectory and localization within the tissue volume.

**Note S2. Experimental paradigm for electrotactile data acquisition in 30 participants.**

**I. Experiments with human subjects**. The experiments with human subjects were performed in compliance with all the ethical regulations under a protocol that was approved by Zhujiang Hospital of Southern Medical University. A total of 30 volunteers participated in this experiment. All of the volunteers gave written informed consent about the experimental procedure. All participants were trained to manipulate the electrotactile system with the help of experimenters until they understood the sensation of electrical stimulation.

**Ⅱ. Body Composition Data Collection**. Participants initially measure their body composition using a body fat scale. A body fat report is printed for each participant for record-keeping purposes. It is important to note that these reports are confidential and must not be disclosed or discussed in public settings.

**III. Perception** **and Pain Threshold Test**. Testing begins with a current amplitude of 0.05mA, incrementally increased to find the perception threshold. The process continues with gradual increases in current until the pain threshold is determined. For subsequent tests, the experimental current is set at half the sum of the perception and pain thresholds.

To ensure perceptual consistency and eliminate potential bias from unequal detectability, we performed individualized threshold calibration under both the inhibitory and non-inhibitory conditions. For each participant, we measured both the perception threshold and pain threshold in each condition. The midpoint of these two thresholds was calculated and used as the stimulation intensity under the corresponding condition. This approach ensured that stimuli were delivered at comparable levels of subjective intensity across conditions, allowing a fair comparison of performance outcomes. Importantly, the inhibitory electrodes were driven with reverse-polarity currents set to one-fourth of the stimulation current. This level is below the perception threshold and does not produce any tactile sensation on its own. Its sole purpose is to spatially constrain current diffusion around the central stimulation electrode, thereby enhancing current focusing and improving spatial precision. This calibration protocol confirms that the observed improvement in pattern recognition accuracy and response time under the inhibitory condition is not attributable to stronger or more easily detectable stimuli, but rather to the reduction of current diffusion and electrode crosstalk achieved by the stimulation–inhibition electrode unit structure.

**IV. Testing with and without Inhibitory Electrodes.**

**Comparative Experiment Setup:**

**Pre-experiment Preparation:** Participants initially experience microcurrent stimulation corresponding to simple line graphics (horizontal, vertical, left diagonal, right diagonal) twice each. This helps participants familiarize themselves with the experimental process and the sensory stimulation.

**With Inhibitory Electrodes Test:** Participants test five different graphic arrangements using the four basic shapes. After receiving the corresponding microcurrent stimulus for each pattern, participants report the perceived graphic, and the system records the reaction time for perception. The arrangements, as shown in Table S1, are tested sequentially, row by row, by the participants. Each stimulation trial delivered a single microcurrent pattern lasting approximately 1 second, followed by a 5-second response window during which participants were required to verbally report the perceived pattern. This time-constrained paradigm was designed to limit deductive reasoning and encourage rapid, perception-driven responses, ensuring that the results primarily reflected real-time tactile discrimination rather than memorization or inference.

**Without Inhibitory Electrodes Test**. Participants repeat the same graphic arrangements to evaluate the differences in stimulation effects when inhibitory electrodes are absent. This part of the experiment aims to compare the clarity and intensity of tactile feedback with and without the use of inhibitory electrodes.

**Data1 Recording and Organization.** All responses, including perceived patterns and reaction times, are recorded and compiled into a dataset. This allows for a direct analysis of the effect of inhibitory electrodes on tactile perception clarity and reaction time.

**Table S1**. Test Sequence for Simple Line Graphic Perception

|  | **1** | **2** | **3** | **4** |
| --- | --- | --- | --- | --- |
|  | **一** | **\|** | **/** | **\** |
|  | **/** | **\|** | **\** | **一** |
| **simple line** | **\|** | **/** | **一** | **\** |
|  | **\** | **一** | **/** | **\|** |
|  | **一** | **/** | **\|** | **\** |

**Ⅴ. Testing Experiment with 10 Pattern Types.**

**Pre-experiment Preparation:** Participants experience microcurrent stimulation for 10 different patterns, with each pattern experienced twice. The patterns are categorized into three types: Simple lines (horizontal, vertical, left diagonal, right diagonal), Geometric shapes (cross, X-shape, square, rectangle), and Complex figures (smiley face, sad face).

**Experiment Procedure:** At the start of the experiment, participants proceed according to the sequence outlined in Table S2. The test involves five patterns per group, with each pair of opposing patterns sequentially numbered from 1 to 10 in Table S2 for the perception tests. Participants are required to choose between two options to identify the pattern they perceive. The sequence and organization in Table S1 facilitate systematic testing and structured response collection, ensuring each participant's response aligns with the standardized experiment design.

**Data2 Recording and Organization.** For each pattern test, responses are recorded along with the corresponding choice (correct or incorrect). These data are organized into a separate dataset that allows for a clear evaluation of recognition accuracy for each pattern type, facilitating later comparisons between pattern groups and conditions.

**Table S2.** Pattern Perception Identification Test Sequence

|  | **1** | **2** | **3** | **4** | **5** | **6** | **7** | **8** | **9** | **10** |
| --- | --- | --- | --- | --- | --- | --- | --- | --- | --- | --- |
| **一 \|** | **一** | **\|** | **\|** | **一** | **一** | **一** | **\|** | **\|** | **\|** | **一** |
| **/ \** | **\** | **/** | **\** | **\** | **/** | **\** | **/** | **/** | **\** | **/** |
| **✕ ✛** | **✕** | **✕** | **✛** | **✛** | **✕** | **✛** | **✕** | **✕** | **✛** | **✛** |
| **□** | **□** |  |  | **□** | **□** |  | **□** | **□** |  |  |
|  |  |  |  |  |  |  |  |  |  |  |

**Note S3. EEG experimental paradigm and analysis for electrotactile stimulation with/without inhibitory electrodes.**

**I. Experimental Paradigm.** In this exploratory EEG experiment, we adopted a within-subject design. Eight healthy right-handed adults, selected from the 30 participants who had completed the main behavioural study, took part in the recording. However, data from two participants were excluded due to excessive artifacts and poor signal quality that left insufficient clean epochs after preprocessing for reliable connectivity estimation; the final EEG sample therefore comprised six participants. All had normal or corrected-to-normal vision, reported no history of neurological or psychiatric disorders, and gave written informed consent prior to participation. Scalp EEG was acquired using an NE Enobio 32 EEG system with electrodes positioned according to the international 10–20 system. Electrical stimulation was delivered to the pulp of the right index fingertip using the same wearable fabric electrotactile device as in the main experiment. Stimulation intensity was individually adjusted for each subject based on their personal sensory threshold, ensuring the stimulus was clearly perceptible without causing significant pain or discomfort. Two stimulation configurations were tested: (1) the stimulation–inhibition configuration with surrounding inhibitory electrodes, and (2) the stimulation-only configuration without inhibitory electrodes. The two conditions were presented in pseudorandom order throughout the session. For each stimulus, a trigger signal was sent simultaneously to the EEG system, and epochs from −0.5 to 3 s relative to the trigger were analysed, covering a pre-stimulus baseline (−0.5–0 s), the brief delay between trigger and effective stimulation onset, and the subsequent cortical response interval. During the experiment, participants sat comfortably in a quiet, dimly lit room, maintained fixation on a central cross, and were instructed to remain relaxed and to minimise eye blinks and body movements; no overt behavioural responses were required.

**Ⅱ. EEG Recording and Processing.** EEG data acquisition was performed using Neuroelectrics' Enobio 32 acquisition device and NIC2 software. Because electrical stimulation of the right index fingertip is expected to elicit the strongest cortical responses over the contralateral (left) primary somatosensory cortex and adjacent sensorimotor regions, we focused on seven electrodes over the left hemisphere and midline: FC5, FC1, Cz, C1, C3, CP1, and P3. These sites cover contralateral precentral/central and paracentral regions corresponding to hand-related sensorimotor representations. Similar contralateral central/paracentral leads have been widely used to capture somatosensory-evoked EEG responses to upper-limb and finger stimulation, particularly over contralateral S1/M1. During online recording, an earlobe electrode served as the reference. Data were sampled at 500 Hz, and an online analog front-end bandpass filter (approximately 2–40 Hz) was applied. Participants were instructed to remain relaxed and to minimise head/body movements and facial muscle tension during recording.

Offline preprocessing was performed in MATLAB using the EEGLAB toolbox. Continuous EEG data were first bandpass filtered using an FIR filter (3–30 Hz). A notch filter at 49–51 Hz was applied to attenuate 50 Hz line noise. To reduce the impact of large artifacts on Independent Component Analysis (ICA), segments containing obvious electrode detachment or gross motion artifacts were removed based on visual inspection. The remaining data were then re-referenced to the average of all recorded electrodes. ICA was subsequently performed, and components associated with eye blinks, eye movements, and muscle activity were identified based on their time courses, power spectra, and scalp topographies and removed. After artifact correction, EEG was epoched from −0.5 to 3s relative to the event marker (time 0), with −0.5 to 0s used as the pre-stimulus baseline. The processed epochs were then used for beta-band functional connectivity strength analysis.

**III. Beta-band functional connectivity computation.** To characterize the influence of the inhibitory electrode on tactile-related cortical networks, this study calculated the correlation coefficients between the beta-band amplitude envelopes of 7 ROI electrodes (FC5, FC1, Cz, C1, C3, P3, CP1) under conditions with (Condition A) and without (Condition B) the inhibitory electrode, serving as indicators of functional connectivity strength.

The specific procedure is as follows: First, apply a 13–30 Hz bandpass filter to the EEG signal $x_{i}(t)$ of each channel to obtain the narrowband beta signal $x_{i}^{\beta}(t)$. Subsequently, a *Hilbert* transform is applied to construct the analytic signal.

$z_{i}(t)=x_{i}^{\beta}(t)+j\mathcal{H}\{x_{i}^{\beta}(t)\}$ (1)

where $\mathcal{H}\{\cdot\}$ denotes the *Hilbert* transform operator. The amplitude envelope is defined as

$A_{i}(t)=\left| z_{i}(t) \right|$ (2)

For any two electrodes $i$ and $j$ ,under given condition $c\in\{A,B\}$ and subject $s$ ,the Pearson correlation coefficient between their amplitude envelope time series $A_{i}^{\left( c,s \right)}(t)$ and $A_{j}^{\left( c,s \right)}(t)$ is taken as the beta functional connectivity strength:

${FC}_{ij}^{\left( \beta\right)}=corr(A_{i}^{\left( c,s \right)}(t),A_{j}^{\left( c,s \right)}(t))$ (3)

For each subject, a $7\times7$ connection matrix ${FC}^{\left( A,s \right)}$ and ${FC}^{\left( B,s \right)}$ was obtained under both inhibited and uninhibited conditions. These matrices were then averaged across subjects to derive the group-level average matrix.

$\overline{FC}_{ij}^{(c)}=\frac{1}{N}\sum_{s=1}^{N} {FC}_{ij}^{(c,s)},c\in\{A,B\}$ (4)

where $N$ denotes the number of subjects ($N=6$ in this study). This average difference matrix between the two conditions is defined as

${\triangle FC}_{\mathrm{ij}}=\overline{FC}_{ij}^{(A)}-\overline{FC}_{ij}^{(B)}$ (5)

**Note S4. Consistency of wire resistance between different devices (Figure 2G).**

Electrode indices S1–S12 correspond to stimulation traces and I1–I12 to inhibition traces. Each coloured curve represents one array. The resistance profiles of all five arrays almost completely overlap across the 24 traces, with values mainly in the range of approximately 10–20 Ω, indicating high device-to-device consistency in both stimulation and inhibition routing.

To assess fabrication uniformity at the device level, we measured the line resistance of all 24 traces (12 stimulation + 12 inhibition) in five independently produced electrode arrays. As shown in Fig. 2G, the resistance pattern as a function of electrode index is highly similar across arrays: local peaks and troughs occur at the same indices for all devices, and the absolute resistance values differ only by a few ohms between arrays. Overall resistance remains within roughly 10–20 Ω for both stimulation (S1–S12) and inhibition (I1–I12) traces.

These results demonstrate that the printed wiring and conductive layers can be reproduced with good uniformity, and that variation between individual devices is small compared with the operating range of the constant-current stimulator. Consequently, differences in behavioural performance measured by TPEIS are unlikely to arise from device-to-device variability in trace resistance.

**Note S5. Electrode–skin impedance measurement and analysis under dry and naturally sweating fingertip conditions.**

Electrode–skin impedance was measured to evaluate the stability of the fabric-based electrode array under dry and naturally sweating fingertip conditions. Eight healthy participants were seated comfortably with the right hand resting on a table. The stimulation–inhibition electrode array (24 conductive traces in total) was mounted on the index fingertip in the same position and orientation as in the main TPEIS experiment, and the elastic band was adjusted to ensure firm but comfortable contact between the electrodes and the skin. Impedance measurements were performed using an ISX-3 electrical impedance spectroscopy system (Impedance Spectroscopy ISX-3, Sciospec, Germany). For each participant, we first acquired impedance data in the dry condition immediately after donning the device. The ISX-3 system sequentially measured the complex impedance at the electrode–skin interface for all 24 traces of the array. From these measurements, the magnitude of the impedance for each trace was extracted, and the 24 values were averaged to obtain a single representative electrode–skin impedance value for that participant in the dry condition.

To obtain the sweating condition, participants then kept the array in place and quietly wore the device for approximately 10 minutes without performing any additional tasks. The firm contact and partial occlusion of the fingertip under the fabric encouraged the natural accumulation of fingertip moisture (sweat). After this period, the impedance measurement procedure was repeated under otherwise identical settings, yielding 24 electrode–skin impedance values per participant for the sweating condition. Again, the mean across traces was used as a representative impedance value for that condition.

For each participant, the value plotted corresponds to the average impedance across all 24 electrode channels on the index fingertip. Blue spheres indicate measurements taken under a dry condition immediately after donning the device, whereas pink pentagrams indicate measurements taken after approximately 10 min of continuous wear, when the fingertip skin had become visibly moist due to perspiration. The impedance values for individual participants remain within a comparable range across conditions (roughly 40–210 kΩ for the dry condition and 20–200 kΩ for the moist condition), with a tendency toward lower impedance after perspiration.

Across the eight participants, fingertip moisture produced by short-term wear leads to a modest, systematic reduction in electrode–skin contact impedance. This trend is consistent with improved ionic conduction in the hydrated stratum corneum, which lowers the effective interface resistance without introducing instability or large between-channel variability. Importantly, even in the lowest-impedance moist condition, all electrode–skin impedances remained within the compliance limits of the constant-current stimulator. The stimulator automatically adjusted its output voltage to maintain the programmed current amplitude, so that the effective charge density at the skin surface was kept under precise control. In practice, this means that moderate sweating does not degrade tactile signal reliability; if anything, the reduced impedance facilitates the generation of perceivable electrotactile sensations at a given current level, supporting the robustness of the system in non-ideal, real-world usage conditions.

**Note S6. Experimental paradigm for Semmes–Weinstein monofilament tactile testing in subjects**

Mechanical detection thresholds were measured using a commercial Semmes–Weinstein monofilament set (Aesthesio®, USA; see Supplementary Fig. S14 for an illustration of the device). Participants were seated comfortably with the right hand resting on a table. Small circular marks were drawn at multiple candidate stimulation sites on the fingers and hand. One of these marks (not disclosed to the participant) was the true target site on the index fingertip. Visual feedback was blocked by an eye mask.

We used a two-alternative forced-choice (2AFC) paradigm. Each trial contained two successive stimulation intervals. In every trial, one of the two intervals always delivered a touch to the index fingertip (target), and the other interval delivered either a touch to a random non-target site (e.g., another phalanx, the palm, or the hand dorsum) or no touch (blank). After each trial, participants were required (i) to indicate whether the first or the second interval contained the touch on the index fingertip, and (ii) to report what happened in the other interval (i.e., which non-target site was touched or that no touch occurred). A trial was scored as correct only if both responses were correct.

A short pre-search phase was used to determine a suprathreshold starting force. The pre-search started at 5.53 g/mm², which was selected as a suprathreshold force level for fingertip stimulation. At this force level, if a participant achieved five consecutive correct trials, the force was accepted as the starting level for the subsequent adaptive procedure. If any incorrect response occurred, the force level was increased by one monofilament step and testing continued until the five-consecutive-correct criterion was met. This pre-search phase was used only to determine the initial suprathreshold starting level and did not contribute to reversal counting.

After determining the starting level, an adaptive staircase was conducted using a 3-down–1-up rule based on the correctness criterion defined above. Specifically, three consecutive correct trials triggered a one-step decrease in monofilament force (down-step), whereas a single incorrect trial triggered a one-step increase (up-step). Within each 2AFC trial, the order of the two intervals was randomized. A reversal was defined as any change in the direction of force adjustment (i.e., up → down or down → up). The staircase continued until six reversals (three reversal pairs) were collected.

The force levels at the last six reversals were averaged to estimate the tactile detection threshold for the index fingertip. Under a 3-down–1-up rule in a 2AFC task, this procedure converges to an estimated detection probability of approximately 79.4%, consistent with standard psychophysical practice.

**Supplementary Movie S1**

In this demo, we show the Tactile Perception Evaluation Interaction System (TPEIS) built on Unity for quantitative evaluation of tactile perception ability in a virtual environment. The system scene is set in a virtual space station, where subjects perceive virtual haptics by touching virtual patterned dots, thus enhancing the fun and immersion of the assessment. At the beginning of the demonstration, a threshold selection interface was shown, where subjects could select the appropriate microcurrent level (1mA, 2mA or 4mA) before the experiment. Subjects wore a VR headset and touched the buttons with their virtual hands to experience the stimulation of different current gears, so that they could choose the most comfortable threshold setting for subsequent experiments. Next, the pre-experimentation phase was demonstrated, in which subjects familiarised themselves with the sensation of tactile perception by experiencing different patterns of microcurrent stimulation. In this phase, a virtual finger generated by the Ultraleap 3Di technology in the VR glasses touched a pattern on the screen, which changed to a lightning symbol when touched, signalling the onset of the microcurrent stimulation. In the pattern recognition task, subjects clicked on an unknown question mark pattern in a virtual box and judged its corresponding pattern type by tactile perception, and the system recorded the result and reaction time of each judgement to further quantify the tactile perception ability. Finally, a tactile perception evaluation report is shown, which is generated based on the subject's judgement results, including the tactile perception score, and provides corresponding suggestions based on the score. If the score is below the lower limit of the standard deviation, the system will suggest to improve the tactile perception ability through repetitive electrical stimulation training.

**Supplementary Movie 2. Virtual Water Flow Interaction**

This movie demonstrates the tactile feedback of warm and hot running water in a virtual kitchen environment. To simulate the gentle flow of water, the electrotactile system uses low-frequency stimulation with moderate pulse width, mimicking the soft sensation of water running over the skin. The low-frequency parameters ensure a smooth, comforting experience, as they are more suited to simulate the mild, continuous touch of water. This design allows users to perceive subtle temperature variations, enhancing the immersion and realism of the interaction.

**Supplementary Movie 3. Virtual Pet Interaction**

In this demo, users interact with a virtual pet by stroking a bird’s forehead. To replicate the delicate sensation of soft feathers, the electrotactile system employs a relatively higher frequency with a short pulse width, providing a light and gentle tactile feedback. While this frequency is higher than the one used for the warm water interaction, it is still lower compared to the cactus interaction. This combination ensures that the feedback feels soft yet distinct, mimicking the subtle but clear sensation of touching a bird’s feathers without being overwhelming.

**Supplementary Movie 4. Virtual Cactus Interaction**

This demonstration simulates the tactile sensation of touching a cactus in a virtual environment. The electrotactile system uses high-frequency stimulation with a short pulse width and high amplitude to replicate the sharp, stinging pain of cactus spines. The high frequency and short pulse width provide a quick and intense response, which accurately mimics the prickling sensation of cactus spines, while the increased amplitude amplifies the intensity of the pain. This design maximizes the realism of the cactus interaction, allowing users to feel a sharp but tolerable sensation.


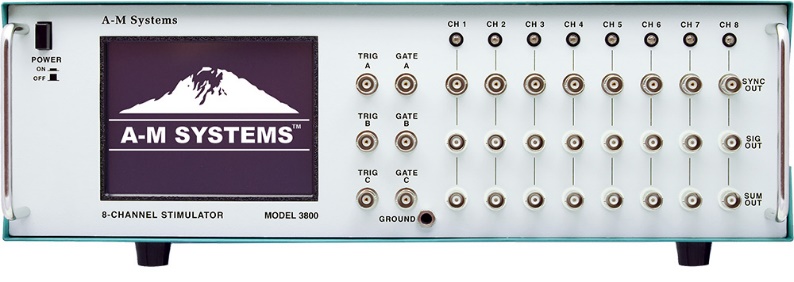


**Fig. S1. Model 3800 MultiStim: 8-Channel Stimulator.** The Model 3800 MultiStim is a high-performance electrophysiological device primarily used in the medical field for electrophysiological research and treatment. It is capable of providing various stimulation modes, including single pulse, dual pulse, and continuous stimulation, and offers adjustable parameters such as frequency and amplitude. The generator has four isolators for converting the pulsed signal into the required stimulus and suppression currents. This versatile device is widely used in fields such as neuromuscular electrophysiological examinations, rehabilitation therapy, and acupuncture. Its features make it highly suitable for electro-tactile feedback applications.


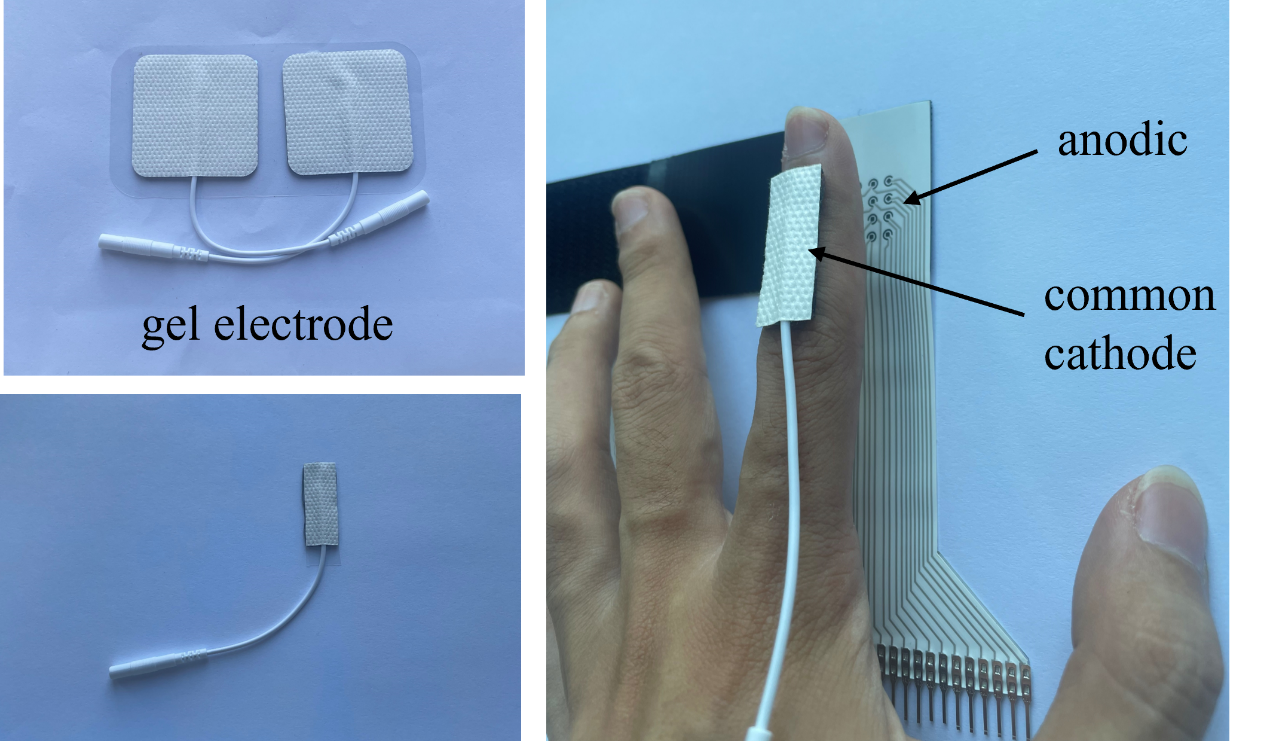


**Fig.** **S2. Optical image schematic of the fingertip-grounding electrode.** The gel electrode is from Shenzhen Baijianda Technology Development Co., Ltd., with product model BJD-B.


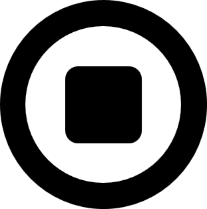


**Fig. S3. Stimulation–inhibition electrode unit structure.** The outer ring electrode serves as the inhibitory electrode, while the central square electrode functions as the stimulating electrode.


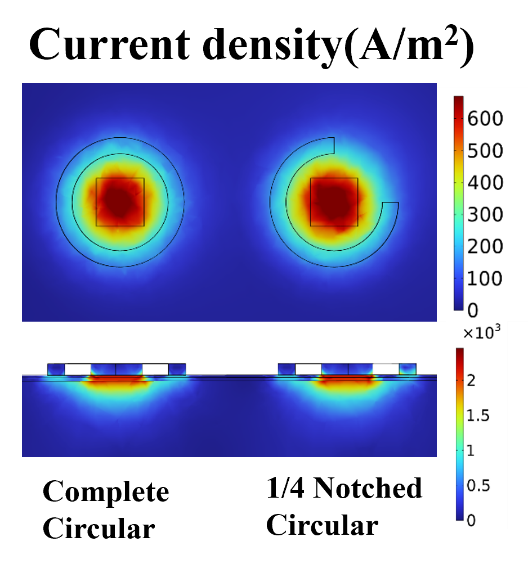


**Fig. S4. Simulation results of current density distribution with and without a 1/4 gap in the ring-shaped electrode.**

**
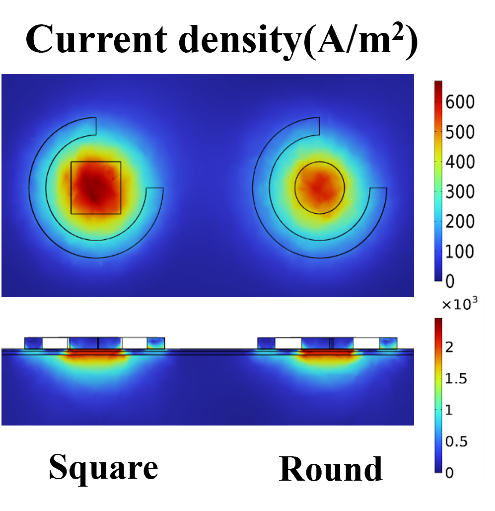
**

**Fig. S5. Simulation results of the current density under different shapes of stimulating electrodes.**


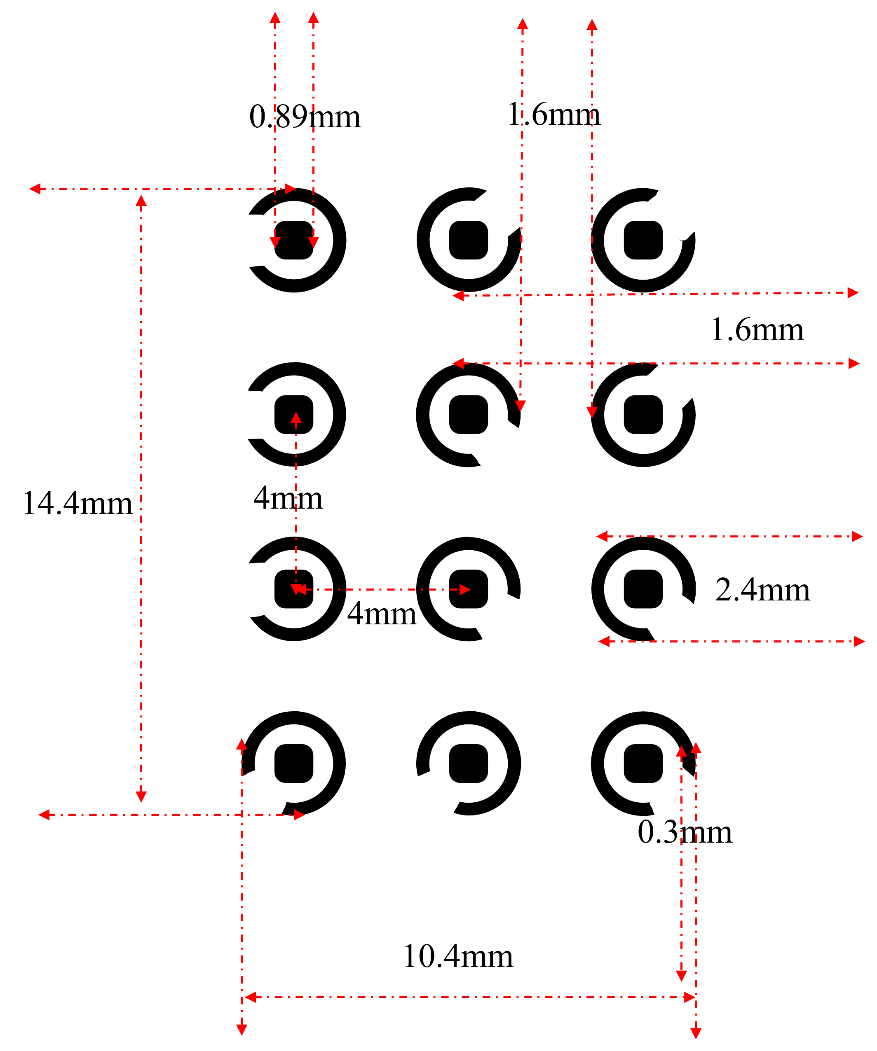


**Fig. S6. Dimensions and Spacing of the Electrode Array.** The electrode array consists of 12 electrodes with a stimulation–inhibition electrode unit structure. In designing the geometry, we aimed to balance two practical requirements: covering the tactile-sensitive area of the fingertip with sufficient channel density, while avoiding excessive current overlap between neighbouring pads that would blur spatial perception. Anatomical studies indicate an effective tactile area of roughly 1–1.5 cm² on the fingertip and a two-point discrimination threshold of about 2–4 mm; accordingly, the overall array footprint was set to 14.4 mm × 10.4 mm, populated by 12 units with a centre-to-centre spacing of 4 mm between stimulating electrodes so that adjacent channels are close enough to render patterns yet remain perceptually separable for most users. Within each unit, the central stimulating electrode is a square structure with a side length of 0.89 mm, chosen as a compromise between spatial precision (smaller pad) and maintaining a low charge density at the selected microcurrent amplitude. The surrounding inhibitory electrode is implemented as a 2.4 mm-diameter ring with a width of 0.3 mm and a insulating gap to the central stimulation electrode. This places the inhibitory conductor sufficiently near the stimulating electrode to “capture” lateral current spread in the near field and reduce crosstalk, while preserving enough gap and ring width for reliable fabrication on the fabric substrate and to avoid short-circuiting under bending. Overall, the final 0.89 mm pad size, 2.4 mm ring, and 4 mm pitch reflect a design trade-off among fingertip anatomy, perceptual separability, safety margins, and manufacturability, rather than arbitrary dimension choices.


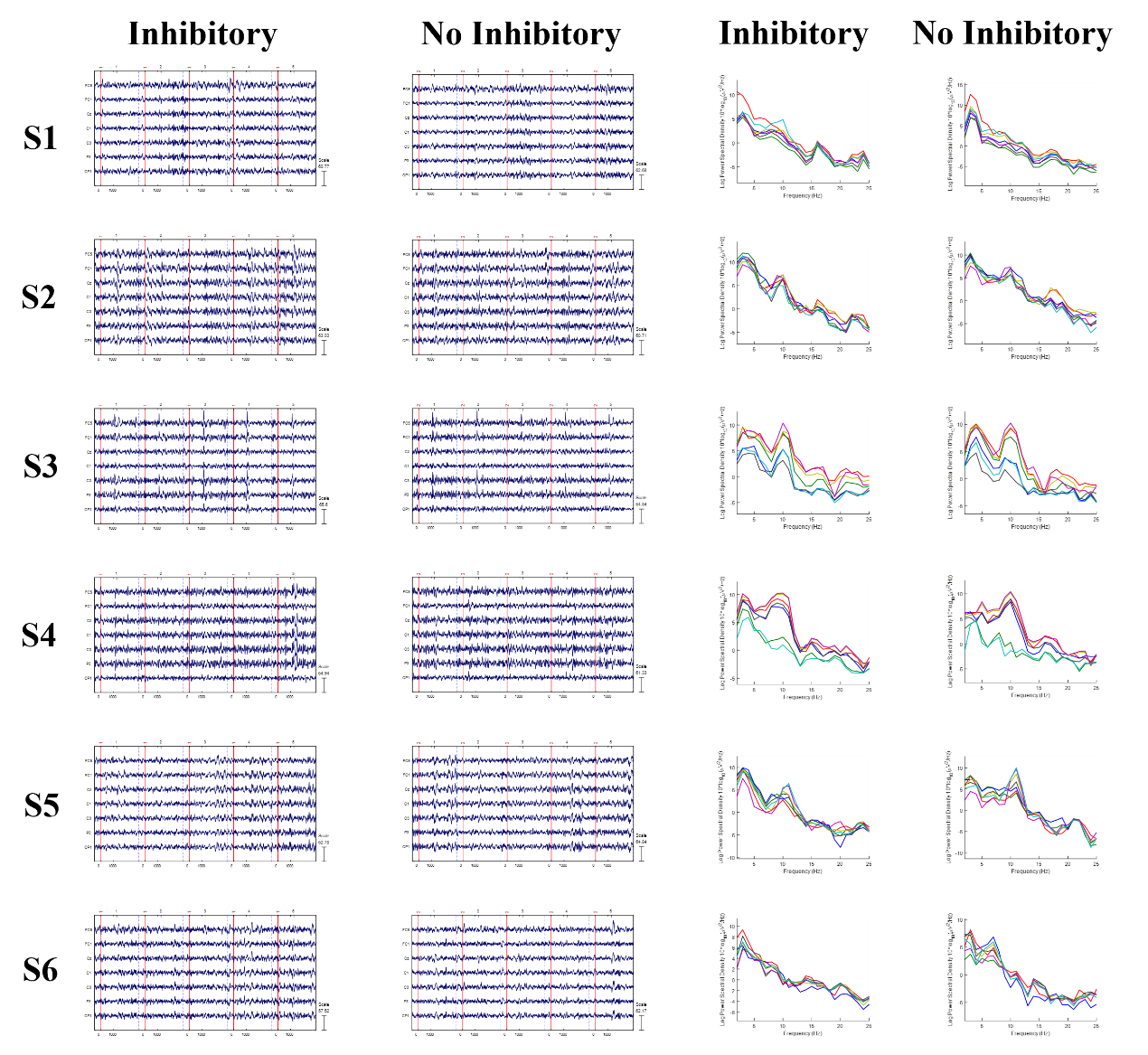


**Fig. S7. Individual EEG traces and power spectra for stimulation with and without inhibitory electrodes.** For each of the six participants (rows), the first and second panels show representative 5s segments of preprocessed EEG recorded from seven electrodes over the left fronto-central and centro-parietal regions (FC5, FC1, C1, C3, Cz, CP1, P3) during electrotactile stimulation with (left) and without (right) inhibitory electrodes. Each trace corresponds to one channel. The third and fourth panels depict the corresponding power spectral density (PSD; 3–30 Hz) averaged across all epochs for the two stimulation configurations; coloured lines indicate different electrodes.


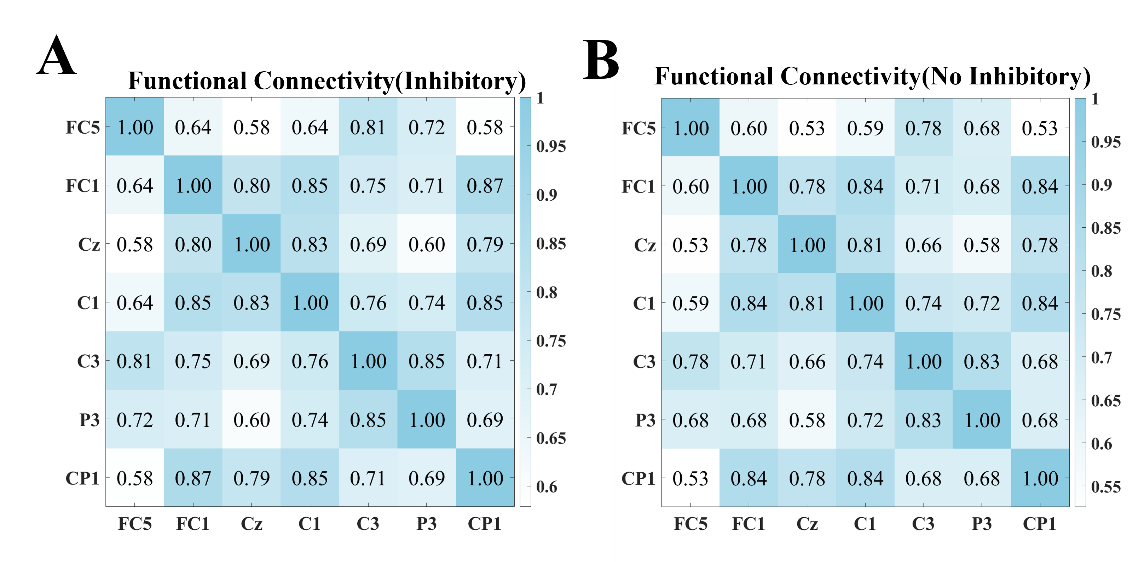


**Fig. S8. Functional connectivity matrix in the beta band for seven channels with and without inhibitory electrodes.** (A) Group-average functional connectivity matrix in the beta band with inhibitory electrodes. (B) Group-average functional connectivity matrix in the beta band without inhibitory electrodes.

Fig. S8A shows the group-averaged beta-band functional connectivity matrix (amplitude-envelope correlations) across the seven ROI electrodes under the inhibitory-electrode condition. As expected, the diagonal elements equal 1.00. Off-diagonal connectivity values are generally high (approximately 0.58–0.87), indicating relatively strong beta-band envelope coupling within this contralateral central–parietal sensorimotor set. Fig. S8B presents the corresponding matrix in the non-inhibitory condition. While the overall spatial pattern is broadly similar, most channel-pair correlations are slightly reduced (approximately 0.53–0.84), indicating that beta functional connectivity within the central-parietal network was generally weaker in the absence of inhibitory electrodes.


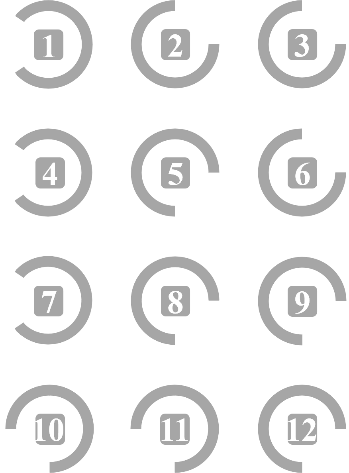


**Fig. S9. Schematic diagram of device electrode numbers.**


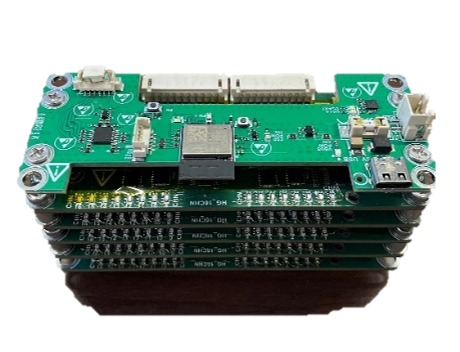


**Fig. S10. Physical Image of the Multi-channel Gate Electrode for Temporal Gating.** The multi-channel gate electrode, which controls the activation state of each channel in the electrode array, is driven by an STM32 microcontroller. By sending control information to the STM32 via Bluetooth, virtual tactile pattern stimuli are rendered on the electrode array while the constant-current stimulator (Model 3800) continuously outputs the programmed pulse train. The gating circuit is based on a microcontroller (MCU) that controls 74HC595 shift registers via an SPI interface to achieve multi-channel expansion and selection functionality. In our implementation, the SPI clock is configured in the MHz range, so shifting and latching a complete pattern word for all channels is completed within several tens of microseconds according to the MCU timing settings and 74HC595 specifications. By incorporating the ULN2803 Darlington array, the output driving capability of the chip is enhanced, enabling precise control of the electrode array through solid-state relays. The propagation delay of the ULN2803–relay stage is on the order of tens to hundreds of microseconds, so the overall channel-switching time from issuing a new gating command on the STM32 to reaching a stable on/off state at the electrodes is well below 1 ms. This is much shorter than the behavioral time scale of pattern presentation (0.1 s per site) and the 2 ms period of the 500 Hz stimulation pulses, making switching latency negligible at the perceptual level and ensuring tight synchronization between VR events and tactile feedback. The system is designed with modularity, using multiple cascaded 74HC595 modules to expand the number of channels. The output signals from the 74HC595 are amplified by the ULN2803 and used to drive the solid-state relays, which control the switching of the electrode array, thus completing the transmission of the selection signal. This design features high scalability, strong driving capability, and high reliability, with the standard header interface allowing for convenient expansion and connection of the electrode array. The overall design ensures the stability and accuracy of the microcurrent tactile feedback system, providing robust hardware support for virtual tactile experiences.


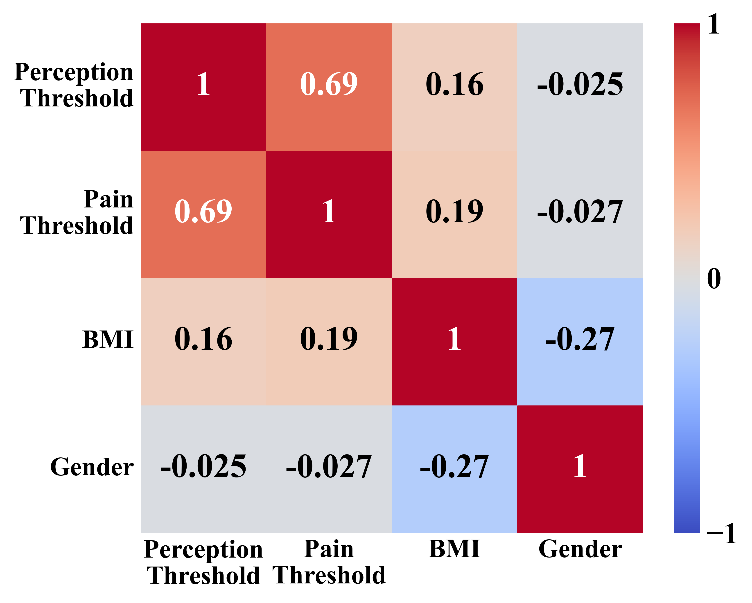


**Fig. S11. Correlation confusion matrix analysis of perception threshold, pain threshold, BMI, and gender.**


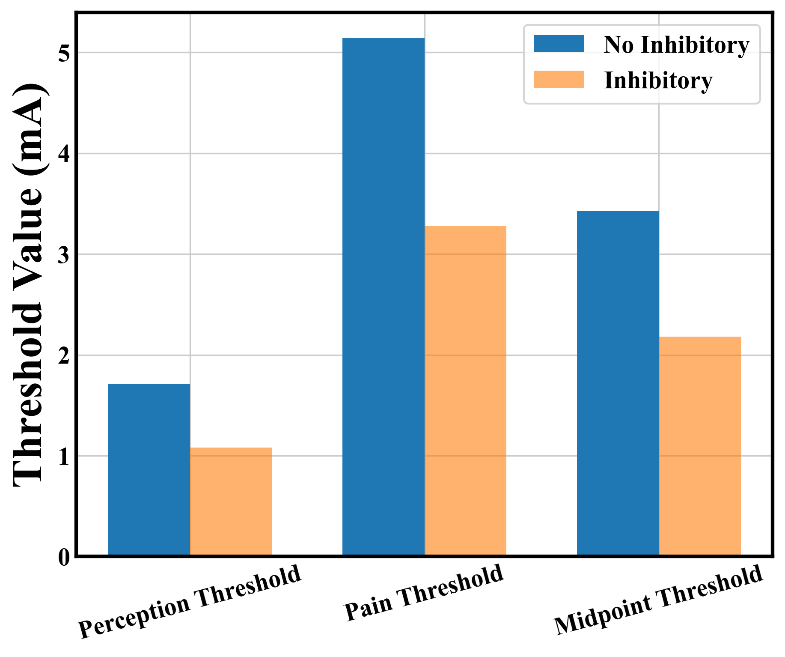


**Fig. S12. Comparative analysis of thresholds with and without inhibitory electrodes.**


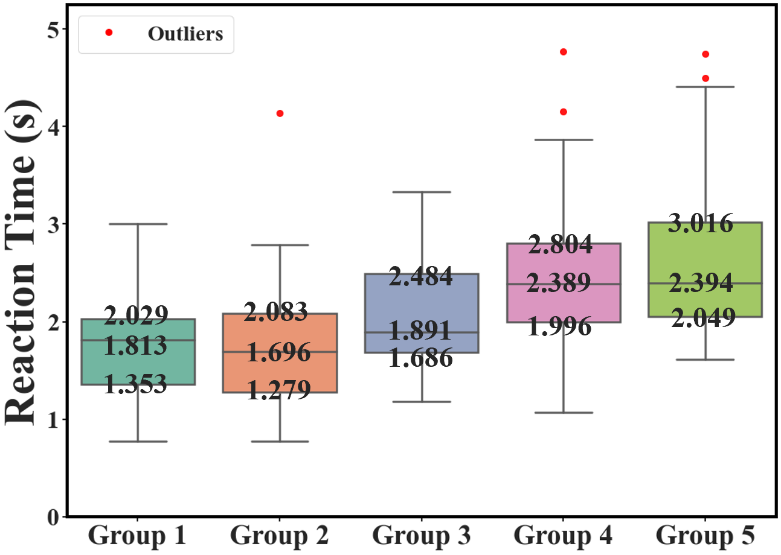


**Fig. S13. Box plots of average reaction time for five groups of tactile patterns among 30 participants.** The plots display the median, interquartile range, and outliers of participant-level mean response times, showing group-dependent variations in processing speed. Group 1 and Group 2 consist of simple stroke patterns (Horizontal and Vertical), while Group 3 and Group 4 represent geometric shapes (Cross, X Shape), and Group 5 represents complex shapes (Smiley Face, Sad Face). The data reveals that as the complexity of the patterns increases, so does the reaction time, with longer median reaction times and wider interquartile ranges in the more complex groups. This suggests that more complex patterns require greater perception processing and result in slower responses.


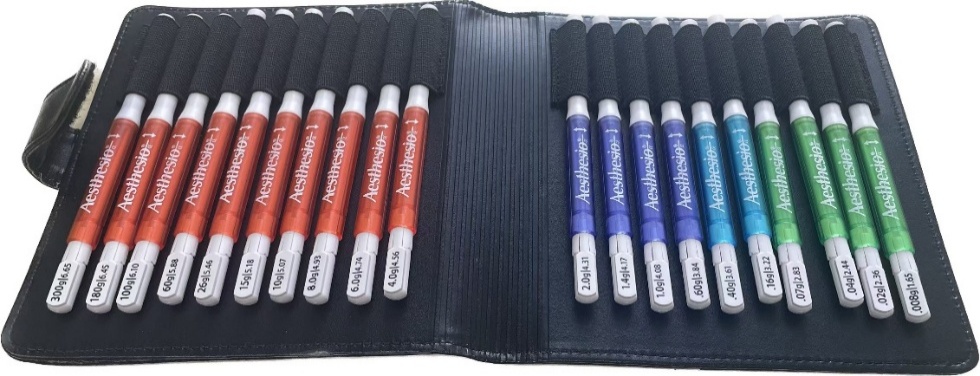


**Fig. S14. Aesthesio® Semmes–Weinstein monofilament set used for mechanical detection tactile threshold measurements.**





**Fig. S15.** **Comparison between mechanical tactile thresholds and TPEIS scores in eight participants.** Pink spheres show the baseline TPEIS scores, and green spheres show the mechanical detection thresholds measured with a Semmes–Weinstein monofilament test at the right index fingertip. Participants who had lower mechanical thresholds (better mechanical sensitivity) tended to obtain higher TPEIS scores, whereas those with higher thresholds performed worse in the electrotactile task. For each of the eight participants, we first obtained a baseline TPEIS score from the VR-based electrotactile pattern recognition task. We then measured the mechanical detection threshold at the right index fingertip using the Semmes–Weinstein monofilament procedure described in Supplementary Note S6. Because higher monofilament thresholds indicate poorer mechanical sensitivity, a negative association was expected if both measures capture related aspects of tactile function. Across participants, mechanical thresholds and TPEIS scores showed a strong negative correlation (Pearson *r* = –0.81, *p* = 0.016), such that mechanically more sensitive individuals tended to perform better in electrotactile discrimination. This provides preliminary convergent validity for the TPEIS metric in this small subset of subjects.





**Fig. S16.** **TPEIS scores of five low-performing participants across one baseline and three post-training tests.** Subjects 1–5 were selected from the main cohort because their baseline TPEIS scores were below one standard deviation of the group mean. The blue line (spheres) shows the baseline (pre-training) score for each subject, and the coloured lines show three repeated tests after a 15-min pattern-based practice in the VR-based electrotactile environment: post-training test 1 (green spheres), post-training test 2 (orange spheres), and post-training test 3 (pink spheres). For all five subjects, post-training scores are consistently higher than baseline, while the three post-training tests cluster within a relatively narrow range for each individual.

From the 30 participants in the main experiment, we first identified five individuals whose initial TPEIS scores were below one standard deviation of the group mean. These low-performing subjects constituted a small cohort for examining short-term practice effects and test–retest reliability. For each subject, we used the original TPEIS measurement as the baseline (Session 0), then provided a 15-min pattern-based practice session with visual feedback in the same VR electrotactile environment, and finally administered three repeated TPEIS tests (Sessions 1–3) under identical task conditions.

As illustrated in Supplementary Fig. S16, all five subjects showed clear score increases from baseline to the first post-training test, indicating that TPEIS is sensitive to short-term improvements after brief task-specific practice. In contrast, scores across the three post-training sessions vary only modestly within each subject, suggesting that once a stable performance level is reached, the TPEIS measure is reasonably reproducible over repeated administrations. Consistent with the main text, we interpret these changes as short-term familiarisation, attentional engagement, and practice-related learning in healthy adults, rather than as evidence of long-term therapeutic or rehabilitative effects.


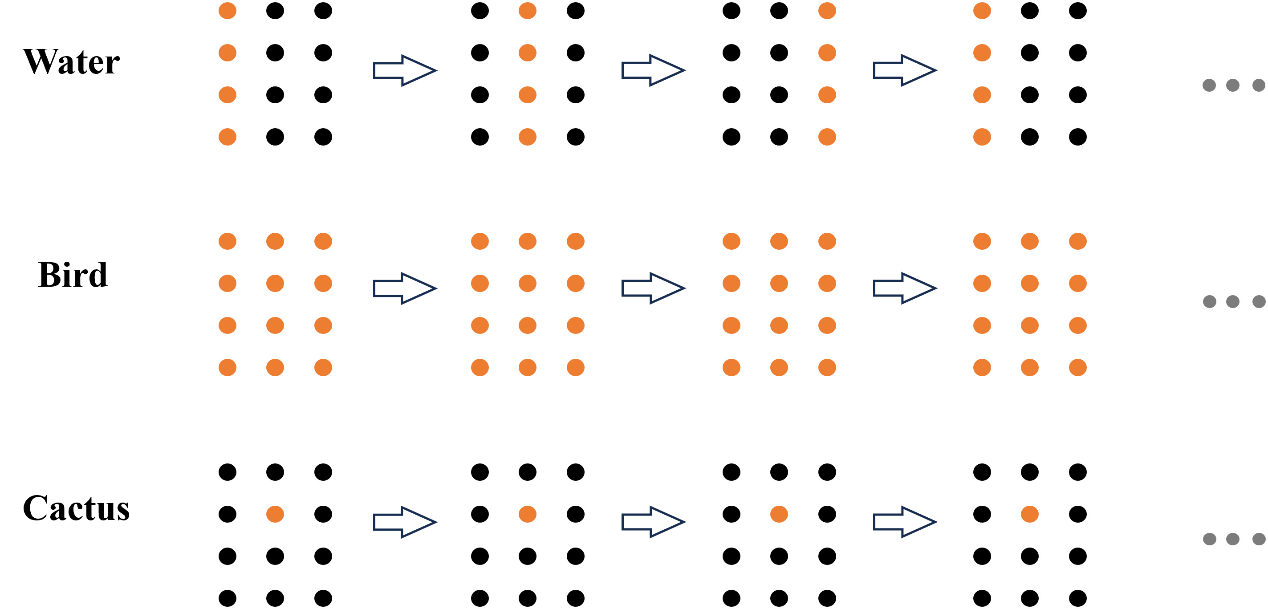


**Fig. S17. Electrode activation sequences and stimulation settings for daily tactile experience scenarios.** For each VR interaction, we co-designed the electrode activation pattern and stimulation waveform based on well-known electrotactile psychophysics: higher frequencies and longer pulse widths tend to produce a more continuous, dense and intense sensation, whereas lower frequencies and shorter pulses are perceived as softer, more discrete “tapping” or gentle vibration; increasing current amplitude primarily scales perceived strength. In the warm-water condition, columns of electrodes are activated sequentially across the array while using a moderate frequency and pulse width (e.g., 200 Hz, 200 µs) and medium current. The column sweep in space gives the impression of motion along the fingertip, and the mid-range temporal parameters yield a smooth, non-irritating vibration, so that users perceive a gentle stream of water. In the hot-water condition, we keep the same column-sweep pattern (so the flow direction is unchanged) but increase frequency and pulse width and slightly raise the current (e.g., 500 Hz, 300 µs). The higher temporal density and charge per pulse make the sensation more continuous and intense, which users interpret—together with the visual steam and colour cues in VR—as hotter water rather than simply “stronger vibration”.

In the bird-stroking scenario, all electrodes are driven simultaneously with an intermediate frequency and short pulse width (e.g., 300 Hz, 100 µs) at relatively low amplitude. Spatially, this full-array activation removes the feeling of individual points and instead produces a broad, uniform contact patch; temporally, the mid-frequency, short-pulse pattern gives a light, fine texture rather than a buzzing or prickling quality, which matches the visual impression of soft feathers. In the cactus scenario, we deliberately choose the opposite corner of the parameter space: a single localized electrode is activated with higher frequency and short pulses at a higher, but still tolerable, current (e.g., 500 Hz, 100 µs). The combination of strong, high-rate stimulation and tight spatial confinement yields a sharp, punctate sensation at one spot, mimicking a cactus spine prick rather than diffuse pressure. During VR operation, when the user’s virtual fingertip contacts the faucet, bird or cactus, the corresponding pre-programmed activation sequence and waveform set are triggered via the multi-channel gating circuit, ensuring that these electrotactile patterns are time-locked to the visual event. The joint mapping of spatial pattern + temporal parameters + visual context is what defines the perceived modality (flowing warm/hot water, soft feathers, or cactus sting).
