# Supplementary material for: Wearable Fabric Electrotactile System with Stimulation–Inhibition Electrode Units
Source: Cyborg Bionic Syst. 2026 Apr 1;7:0515. doi: 10.34133/cbsystems.0515 (PMC13039521; doi:10.34133/cbsystems.0515)
Supplement: Supplementary 1 — Notes S1 to S6 Movies S1 to S4 Figs. S1 to S17 Tables S1 and S2 [file cbsystems.0515.f1.zip › supplementary information.pdf]

Supplementary Information for

Wearable Fabric Electrotactile System with Stimulation−Inhibition Electrode Units

Hongbo Yao *et al.*

Corresponding authors: Lin Shu: shul@scut.edu.cn, Lei Wei: lei.wei@deakin.edu.au ,
Xinge Yu: xingeyu@cityu.edu.hk, Xiangmin Xu: xmxu@scut.edu.cn

This PDF file includes:

Supplementary Notes S1 to S2
Captions for Supplementary Movies 1 to 4
Supplementary Figs. S1 to S11
Supplementary Tables S1 to S2

Other Supplementary Materials for this manuscript include the following:

Supplementary Movies S1 to S4

Note S1. Mechanism of the stimulation–inhibition electrode unit.

To improve the spatial resolution of electrotactile stimulation, we designed a stimulation–inhibition electrode unit structure in which a low-amplitude reverse-polarity current is applied to the peripheral electrodes surrounding the central stimulation site. This approach aims to mitigate the lateral current diffusion typically observed in multi-electrode arrays due to the heterogeneous conductivity and dielectric characteristics of the skin. In conventional configurations, electrical current tends to spread laterally across the skin and subdermal layers, resulting in a dispersed and blurred current density profile. This dispersion may lead to unintended stimulation of adjacent areas and reduce the precision of activating specific mechanoreceptors such as Meissner corpuscles or Merkel cells, thereby degrading tactile localization and recognition performance.

The stimulation–inhibition electrode unit introduces a low-potential electric boundary around the central electrode. By applying a reverse-polarity current (typically one-fourth of the main stimulation amplitude) to the surrounding electrodes, an opposing electric field is generated. This field redistributes the spatial potential landscape, effectively forming an "electric potential fence" that suppresses outward current leakage and enhances vertical penetration toward target receptors. As demonstrated in Figure 2a of the main text, we conducted finite element simulations of current density distributions under varying inhibitory-to-stimulation current ratios (0, 1/8, 1/4, 1/2, 3/4, and 1). When no inhibitory current is applied, current disperses widely, and the high-density region is poorly defined. Introducing a moderate inhibitory current (e.g., 1/8 or 1/4) results in a more focused and intensified current peak directly beneath the stimulating electrode. The 1:4 ratio yields the most compact and intense central focus, minimizing peripheral activation. However, increasing the inhibitory current beyond this optimal point causes the surrounding electrodes to generate sufficient reverse current to themselves activate peripheral mechanoreceptors. This may lead to multi-point perceptual confusion or sensory numbness, counteracting the intended focusing effect. Therefore, we define the 1:4 ratio as a safe and effective balance between spatial precision and minimal off-target stimulation.

This mechanism provides theoretical support for the enhanced performance observed in our pattern recognition experiments under the inhibitory condition. It also confirms that the stimulation–inhibition electrode unit structure design enhances spatial tactile resolution not by increasing current strength, but by controlling current trajectory and localization within the tissue volume.

Note S2. Experimental paradigm for electrotactile data acquisition in 30 participants.

I. Experiments with human subjects. The experiments with human subjects were performed in compliance with all the ethical regulations under a protocol that was approved by Zhujiang Hospital of Southern Medical University. A total of 30 volunteers participated in this experiment. All of the volunteers gave written informed consent about the experimental procedure. All participants were trained to manipulate the electrotactile system with the help of experimenters until they understood the sensation of electrical stimulation.

II. Body Composition Data Collection. Participants initially measure their body composition using a body fat scale. A body fat report is printed for each participant for record-keeping purposes. It is important to note that these reports are confidential and must not be disclosed or discussed in public settings.

III. Perception and Pain Threshold Test. Testing begins with a current amplitude of 0.05mA, incrementally increased to find the perception threshold. The process continues with gradual increases in current until the pain threshold is determined. For subsequent tests, the experimental current is set at half the sum of the perception and pain thresholds.

To ensure perceptual consistency and eliminate potential bias from unequal detectability, we performed individualized threshold calibration under both the inhibitory and non-inhibitory conditions. For each participant, we measured both the perception threshold and pain threshold in each condition. The midpoint of these two thresholds was calculated and used as the stimulation intensity under the corresponding condition. This approach ensured that stimuli were delivered at comparable levels of subjective intensity across conditions, allowing a fair comparison of performance outcomes. Importantly, the inhibitory electrodes were driven with reverse-polarity currents set to one-fourth of the stimulation current. This level is below the perception threshold and does not produce any tactile sensation on its own. Its sole purpose is to spatially constrain current diffusion around the central stimulation electrode, thereby enhancing current focusing and improving spatial precision. This calibration protocol

confirms that the observed improvement in pattern recognition accuracy and response time under the inhibitory condition is not attributable to stronger or more easily detectable stimuli, but rather to the reduction of current diffusion and electrode crosstalk achieved by the stimulation–inhibition electrode unit structure.

IV. Testing with and without Inhibitory Electrodes.

Comparative Experiment Setup:

Pre-experiment Preparation: Participants initially experience microcurrent stimulation corresponding to simple line graphics (horizontal, vertical, left diagonal, right diagonal) twice each. This helps participants familiarize themselves with the experimental process and the sensory stimulation.

With Inhibitory Electrodes Test: Participants test five different graphic arrangements using the four basic shapes. After receiving the corresponding microcurrent stimulus for each pattern, participants report the perceived graphic, and the system records the reaction time for perception. The arrangements, as shown in Table S1, are tested sequentially, row by row, by the participants. Each stimulation trial delivered a single microcurrent pattern lasting approximately 1 second, followed by a 5-second response window during which participants were required to verbally report the perceived pattern. This time-constrained paradigm was designed to limit deductive reasoning and encourage rapid, perception-driven responses, ensuring that the results primarily reflected real-time tactile discrimination rather than memorization or inference.

Without Inhibitory Electrodes Test. Participants repeat the same graphic arrangements to evaluate the differences in stimulation effects when inhibitory electrodes are absent. This part of the experiment aims to compare the clarity and intensity of tactile feedback with and without the use of inhibitory electrodes.

Data1 Recording and Organization. All responses, including perceived patterns and reaction times, are recorded and compiled into a dataset. This allows for a direct analysis of the effect of inhibitory electrodes on tactile perception clarity and reaction time.

Table S1. Test Sequence for Simple Line Graphic Perception

<table>
<tr><th></th><th>1</th><th>2</th><th>3</th><th>4</th></tr>
<tr><td rowspan="5">simple line</td><td>—</td><td>|</td><td>/</td><td>\</td></tr>
<tr><td>/</td><td>|</td><td>\</td><td>—</td></tr>
<tr><td>|</td><td>/</td><td>—</td><td>\</td></tr>
<tr><td>\</td><td>—</td><td>/</td><td>|</td></tr>
<tr><td>—</td><td>/</td><td>|</td><td>\</td></tr>
</table>

V. Testing Experiment with 10 Pattern Types.

Pre-experiment Preparation: Participants experience microcurrent stimulation for 10 different patterns, with each pattern experienced twice. The patterns are categorized into three types: Simple lines (horizontal, vertical, left diagonal, right diagonal), Geometric shapes (cross, X-shape, square, rectangle), and Complex figures (smiley face, sad face).

Experiment Procedure: At the start of the experiment, participants proceed according to the sequence outlined in Table S2. The test involves five patterns per group, with each pair of opposing patterns sequentially numbered from 1 to 10 in Table S2 for the perception tests. Participants are required to choose between two options to identify the pattern they perceive. The sequence and organization in Table S1 facilitate systematic testing and structured response collection, ensuring each participant's response aligns with the standardized experiment design.

Data2 Recording and Organization. For each pattern test, responses are recorded along with the corresponding choice (correct or incorrect). These data are organized into a separate dataset that allows for a clear evaluation of recognition accuracy for each pattern type, facilitating later comparisons between pattern groups and conditions.

Table S2. Pattern Perception Identification Test Sequence

	**1**	**2**	**3**	**4**	**5**	**6**	**7**	**8**	**9**	**10**
— ∣	—	∣	∣	—	—	—	∣	∣	∣	—
/ \	\	/	\	\	/	\	/	/	\	/
× +	×	×	+	+	×	+	×	×	+	+
□ ▯	□	▯	▯	□	□	▯	□	□	▯	▯
☺ ☹	☺	☹	☹	☺	☹	☹	☺	☺	☺	☹

Supplementary Movie S1

In this demo, we show the Tactile Perception Evaluation Interactive System (TPEIS) built on Unity for quantitative evaluation of tactile perception ability in a virtual environment. The system scene is set in a virtual space station, where subjects perceive virtual haptics by touching virtual patterned dots, thus enhancing the fun and immersion of the assessment. At the beginning of the demonstration, a threshold selection interface was shown, where subjects could select the appropriate microcurrent level (1mA, 2mA or 4mA) before the experiment. Subjects wore a VR headset and touched the buttons with their virtual hands to experience the stimulation of different current gears, so that they could choose the most comfortable threshold setting for subsequent experiments. Next, the pre-experimentation phase was demonstrated, in which subjects familiarised themselves with the sensation of tactile perception by experiencing different patterns of microcurrent stimulation. In this phase, a virtual finger generated by the Ultraleap 3Di technology in the VR glasses touched a pattern on the screen, which changed to a lightning symbol when touched, signalling the onset of the microcurrent stimulation. In the pattern recognition task, subjects clicked on an unknown question mark pattern in a virtual box and judged its corresponding pattern type by tactile perception, and the system recorded the result and reaction time of each judgement to further quantify the tactile perception ability. Finally, a tactile perception evaluation report is shown, which is generated based on the subject's judgement results, including the tactile perception score, and provides corresponding suggestions based on the score. If the score is below the lower limit of the standard deviation, the system will suggest to improve the tactile perception ability through repetitive electrical stimulation training.

Supplementary Movie 2. Virtual Water Flow Interaction

This movie demonstrates the tactile feedback of warm and hot running water in a virtual kitchen environment. To simulate the gentle flow of water, the electrotactile system uses low-frequency stimulation with moderate pulse width, mimicking the soft sensation of water running over the skin. The low-frequency parameters ensure a smooth, comforting experience, as they are more suited to simulate the mild, continuous touch of water. This design allows users to perceive subtle temperature variations, enhancing the immersion and realism of the interaction.

Supplementary Movie 3. Virtual Pet Interaction

In this demo, users interact with a virtual pet by stroking a bird’s forehead. To replicate the delicate sensation of soft feathers, the electrotactile system employs a relatively higher frequency with a short pulse width, providing a light and gentle tactile feedback. While this frequency is higher than the one used for the warm water interaction, it is still lower compared to the cactus interaction. This combination ensures that the feedback feels soft yet distinct, mimicking the subtle but clear sensation of touching a bird’s feathers without being overwhelming.

Supplementary Movie 4. Virtual Cactus Interaction

This demonstration simulates the tactile sensation of touching a cactus in a virtual environment. The electrotactile system uses high-frequency stimulation with a short pulse width and high amplitude to replicate the sharp, stinging pain of cactus spines. The high frequency and short pulse width provide a quick and intense response, which accurately mimics the prickling sensation of cactus spines, while the increased amplitude amplifies the intensity of the pain. This design maximizes the realism of the cactus interaction, allowing users to feel a sharp but tolerable sensation.

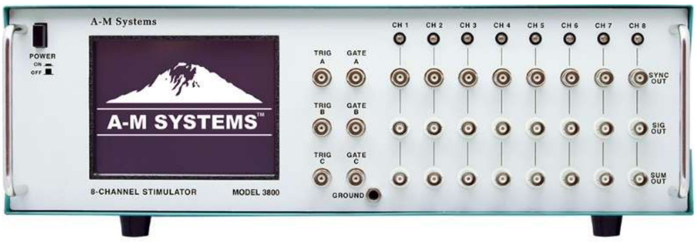

Fig. S1. Model 3800 MultiStim: 8-Channel Stimulator. The Model 3800 MultiStim is a high-performance electrophysiological device primarily used in the medical field for electrophysiological research and treatment. It is capable of providing various stimulation modes, including single pulse, dual pulse, and continuous stimulation, and offers adjustable parameters such as frequency and amplitude. The generator has four isolators for converting the pulsed signal into the required stimulus and suppression currents. This versatile device is widely used in fields such as neuromuscular electrophysiological examinations, rehabilitation therapy, and acupuncture. Its features make it highly suitable for electro-tactile feedback applications.

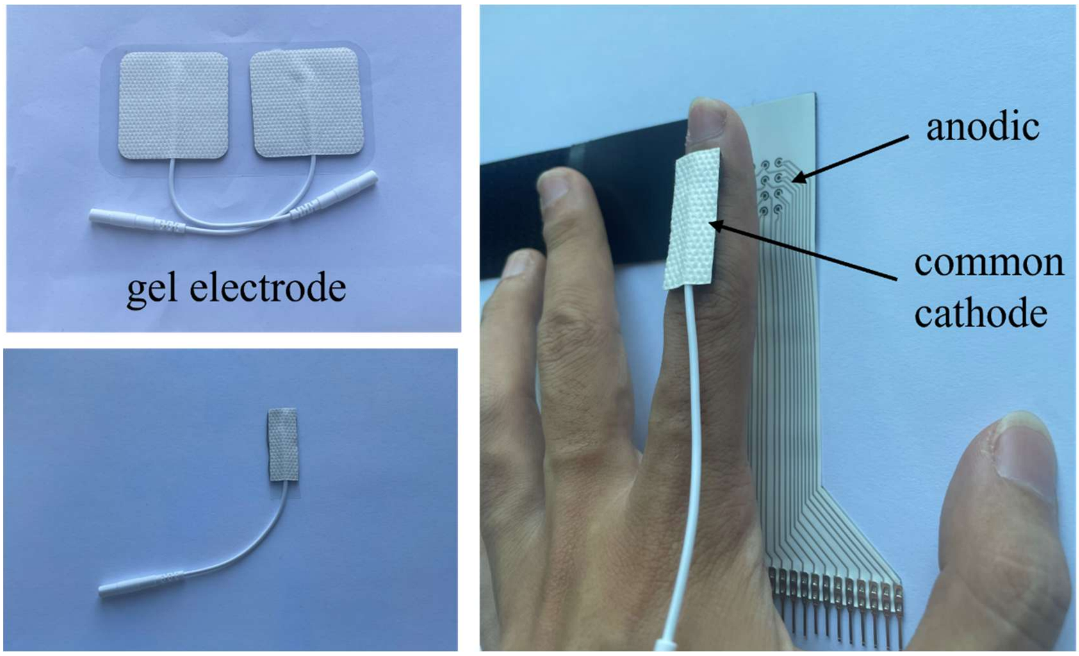

Fig. S**2. Optical image schematic of the fingertip-grounding electrode.** The gel electrode is from Shenzhen Baijianda Technology Development Co., Ltd., with product model BJD-B.

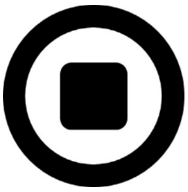

Fig. S3. Stimulation–inhibition electrode unit structure. The outer ring electrode serves as the inhibitory electrode, while the central square electrode functions as the stimulating electrode.

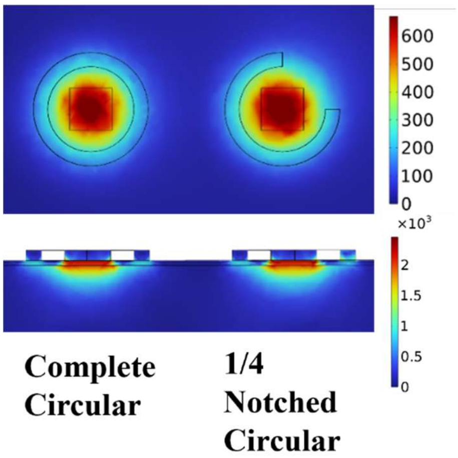

Fig. S4. Simulation results of current density distribution with and without a 1/4 gap in the ring-shaped electrode.

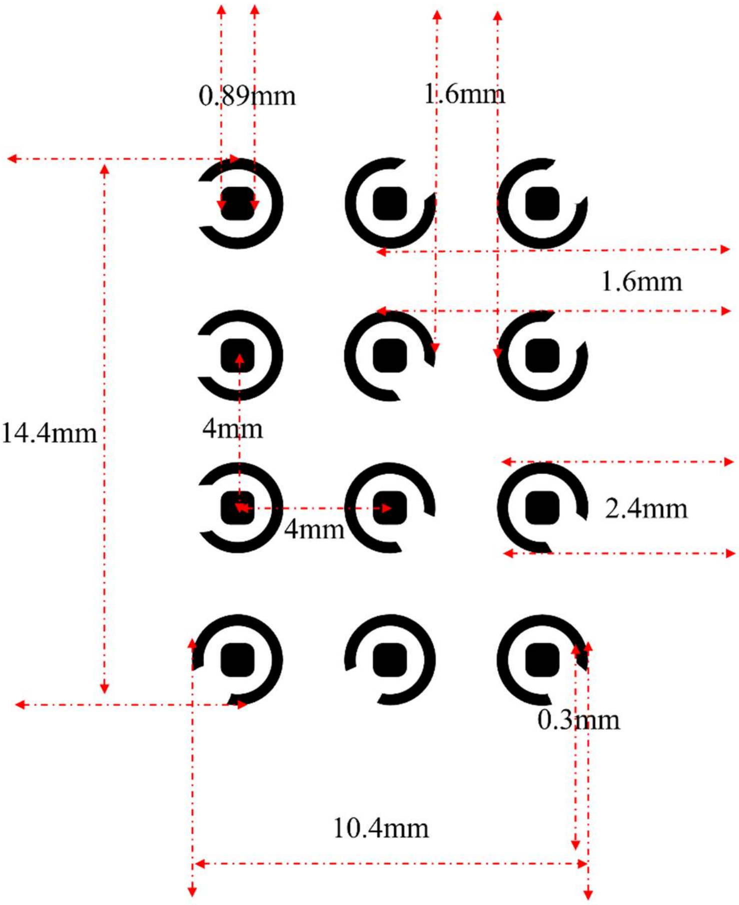

Fig. S5. Dimensions and Spacing of the Electrode Array. The electrode array consists of 12 electrodes with a stimulation–inhibition electrode unit structure. Each surrounding inhibitory electrode is designed as a ring with a diameter of 2.4 mm, where the width of the inhibitory electrode ring is 0.3 mm, and the central stimulating electrode is a square structure with a side length of 0.89 mm. The spacing between the surrounding inhibitory electrodes is 1.6 mm, while the distance between the centers of two stimulating electrodes is 4 mm, which aligns with the typical two-point tactile threshold range for human fingertips (2-4 mm). The total size of the electrode array is 14.4 mm × 10.4 mm, which sufficiently covers the average tactile sensitive area of the general population (approximately 1-1.5 cm²).

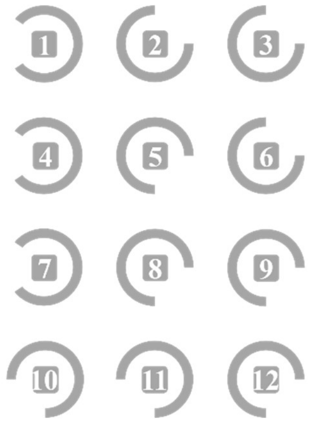

Fig. S6. Schematic diagram of device electrode numbers.

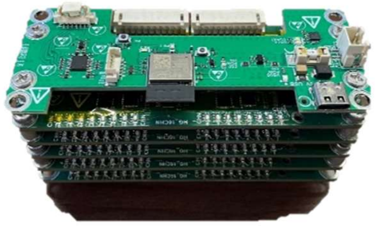

Fig. S7. Physical Image of the Multi-channel Gate Electrode for Temporal Gating. The multi-channel gate electrode, which controls the activation of electrodes, is controlled by STM32. By sending control information to the STM32 via Bluetooth, virtual tactile pattern stimuli can be generated on the electrode array. The gating circuit is based on a microcontroller (MCU) that controls the 74HC595 shift register via an SPI interface to achieve multi-channel expansion and selection functionality. By incorporating the ULN2803 Darlington array, the output driving capability of the chip is enhanced, enabling precise control of the electrode array through solid-state relays. The system is designed with modularity, using multiple cascaded 74HC595 modules to expand the number of channels. The output signals from the 74HC595 are amplified by the ULN2803 and used to drive the solid-state relays, which control the switching of the electrode array, thus completing the transmission of the selection signal. This design features high scalability, strong driving capability, and high reliability, with the standard header interface allowing for convenient expansion and connection of the electrode array. The overall design ensures the stability and accuracy of the microcurrent tactile feedback system, providing robust hardware support for virtual tactile experiences.

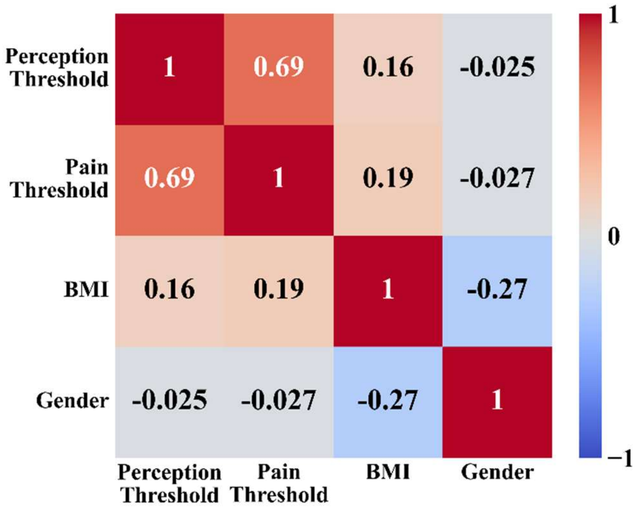

Fig. S8. Correlation confusion matrix analysis of perception threshold, pain threshold, BMI, and gender.

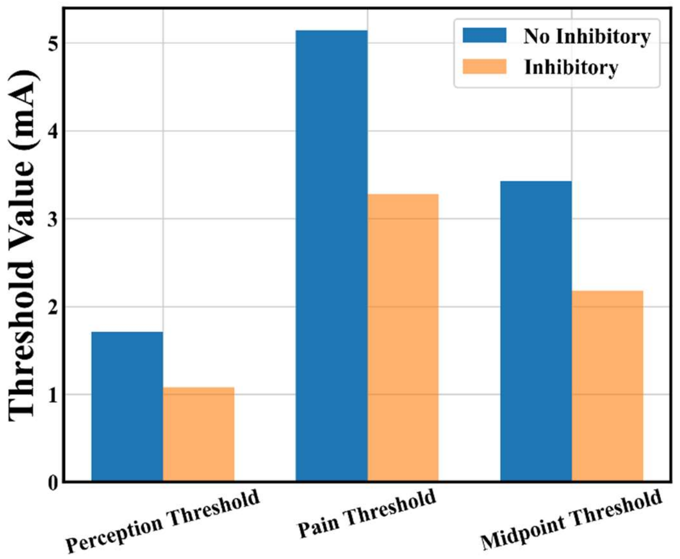

Fig. S9. Comparative analysis of thresholds with and without inhibitory electrodes.

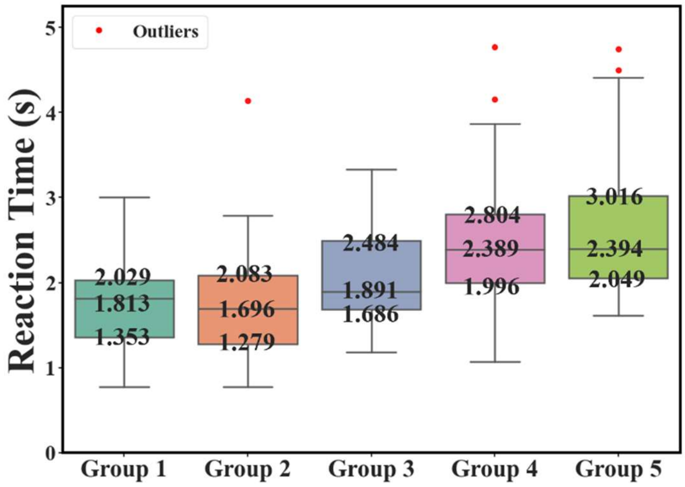

Fig. S10. Box plots of average reaction time for five groups of tactile patterns among 30 participants. The plots display the median, interquartile range, and outliers of participant-level mean response times, showing group-dependent variations in processing speed. Group 1 and Group 2 consist of simple stroke patterns (Horizontal and Vertical), while Group 3 and Group 4 represent geometric shapes (Cross, X Shape), and Group 5 represents complex shapes (Smiley Face, Sad Face). The data reveals that as the complexity of the patterns increases, so does the reaction time, with longer median reaction times and wider interquartile ranges in the more complex groups. This suggests that more complex patterns require greater perception processing and result in slower responses.

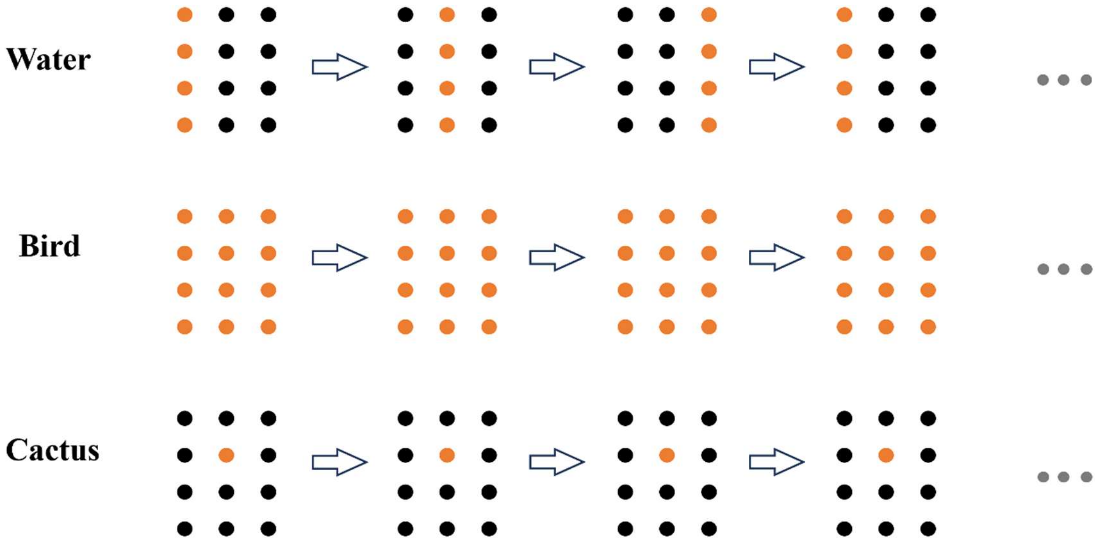

Fig. S11. Electrode activation sequences and stimulation settings for daily tactile experience scenarios. Electrode activation sequences corresponding to different VR interaction scenarios are illustrated. In the virtual kitchen task, sequential activation of electrode columns induces a continuous flow sensation to simulate running water. During the bird interaction, all electrode channels are simultaneously activated to provide uniform and delicate tactile feedback, mimicking the sensation of stroking feathers. In the cactus scenario, localized single-electrode stimulation evokes a sharp stinging sensation, reproducing the effect of fingertip contact with cactus spines. These spatiotemporal activation strategies illustrate how the coordination between electrode gating and stimulation parameters enables differentiated tactile experiences across diverse immersive scenarios.
