# Supplementary figures and images for: Wearable Fabric Electrotactile System with Stimulation–Inhibition Electrode Units
Source: Cyborg Bionic Syst. 2026 Apr 1;7:0515. doi: 10.34133/cbsystems.0515 (PMC13039521; doi:10.34133/cbsystems.0515)

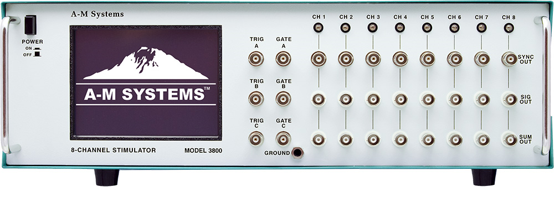

Supplement: Supplementary 1 — Notes S1 to S6 Movies S1 to S4 Figs. S1 to S17 Tables S1 and S2 [file cbsystems.0515.f1.zip › Supplementary Fig. S1.tif]

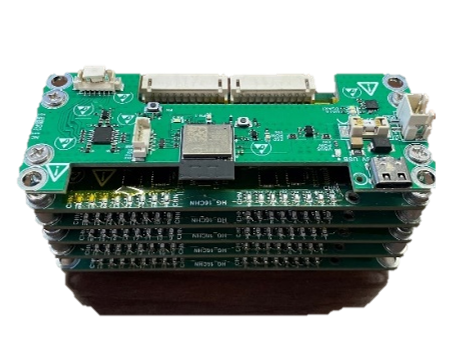

Supplement: Supplementary 1 — Notes S1 to S6 Movies S1 to S4 Figs. S1 to S17 Tables S1 and S2 [file cbsystems.0515.f1.zip › Supplementary Fig. S10.tif]

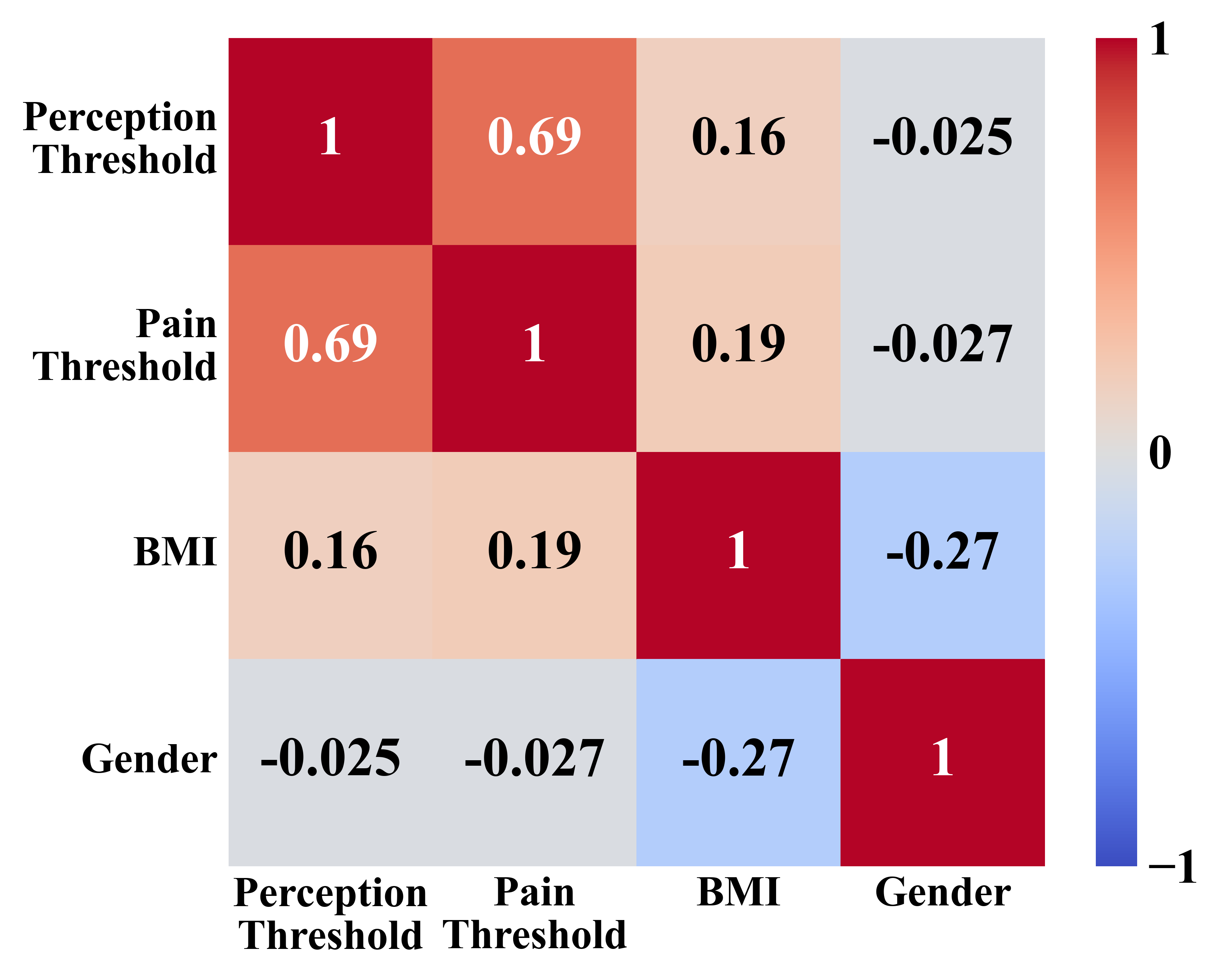

Supplement: Supplementary 1 — Notes S1 to S6 Movies S1 to S4 Figs. S1 to S17 Tables S1 and S2 [file cbsystems.0515.f1.zip › Supplementary Fig. S11.tif]

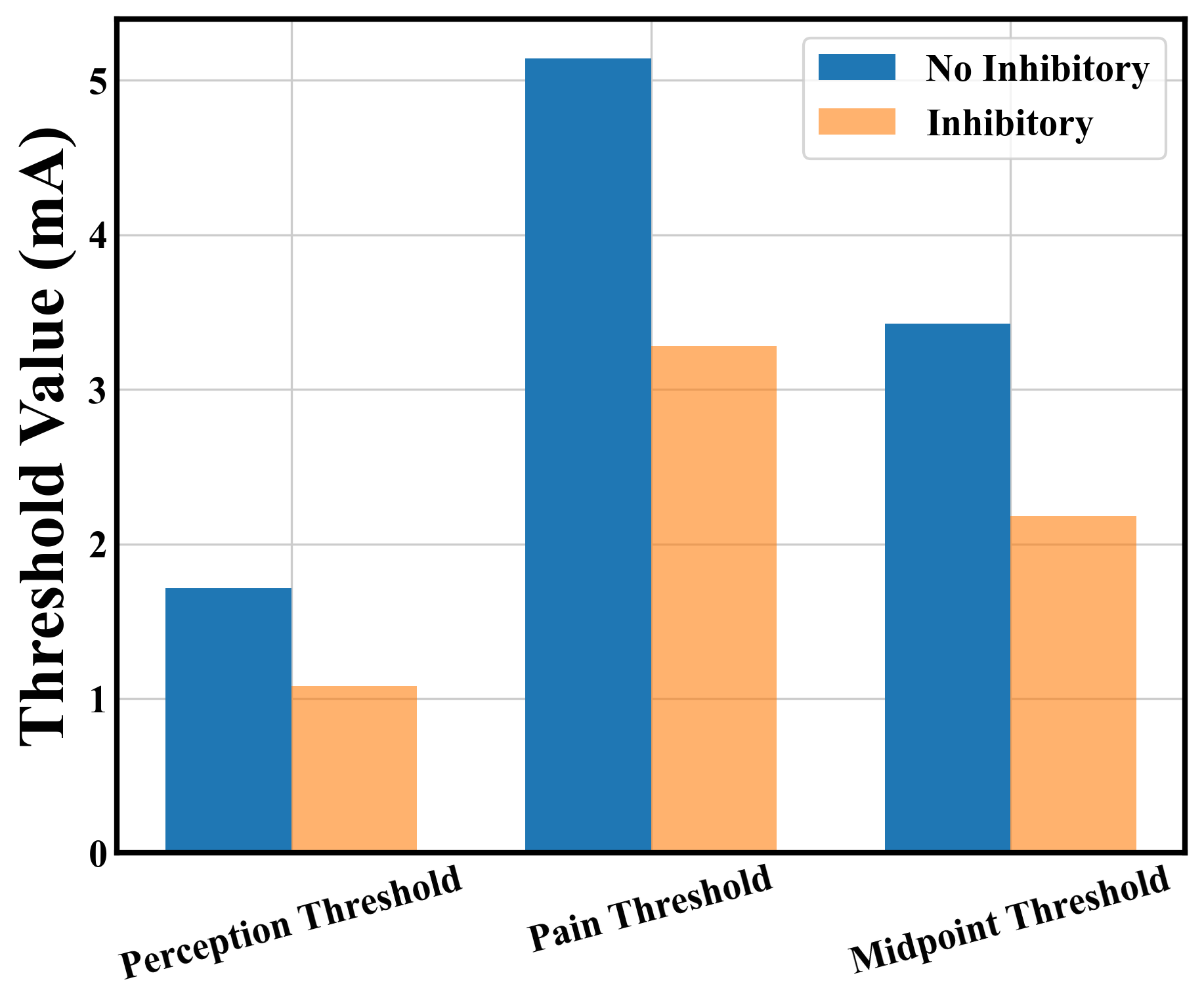

Supplement: Supplementary 1 — Notes S1 to S6 Movies S1 to S4 Figs. S1 to S17 Tables S1 and S2 [file cbsystems.0515.f1.zip › Supplementary Fig. S12.tif]

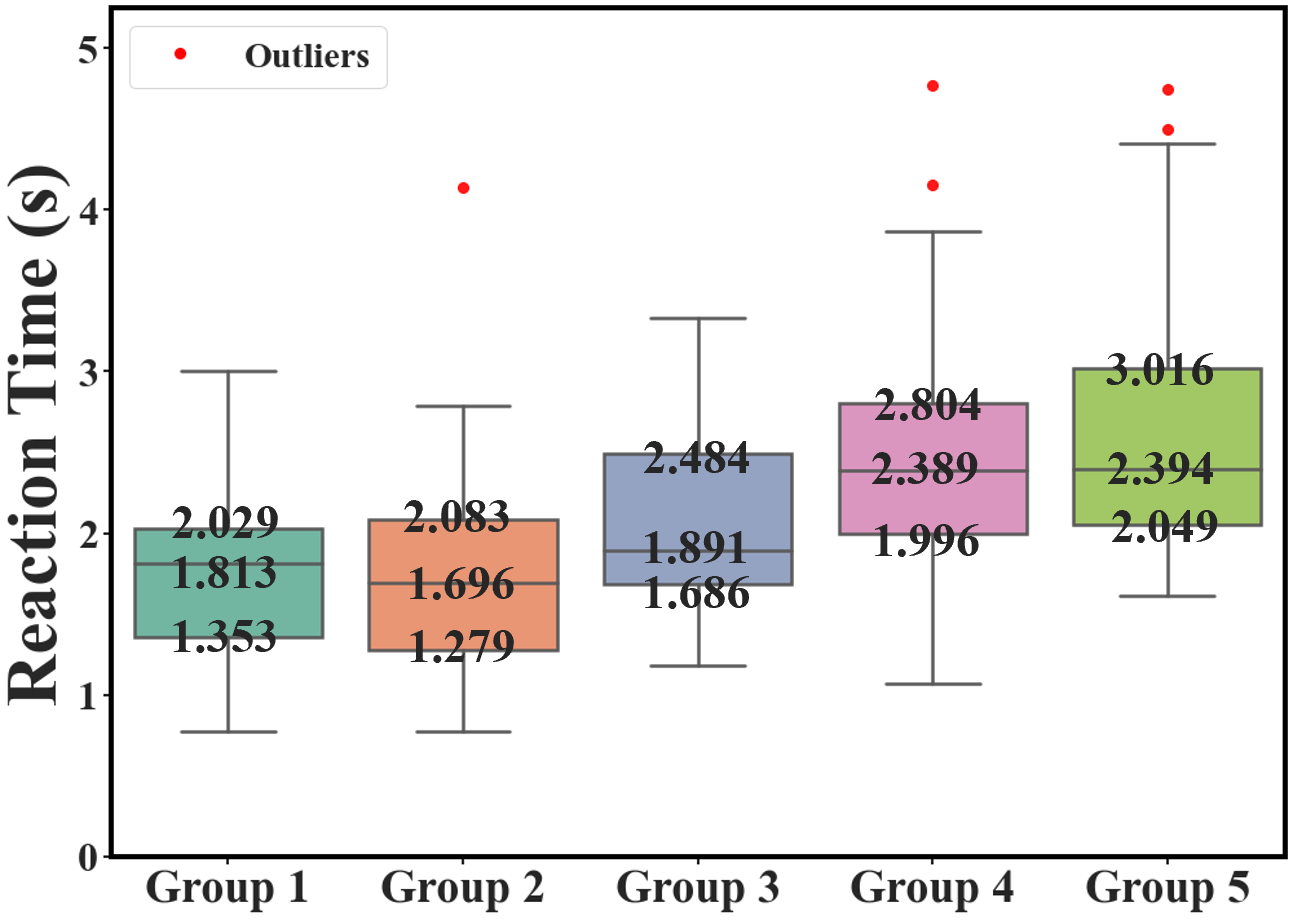

Supplement: Supplementary 1 — Notes S1 to S6 Movies S1 to S4 Figs. S1 to S17 Tables S1 and S2 [file cbsystems.0515.f1.zip › Supplementary Fig. S13.tif]

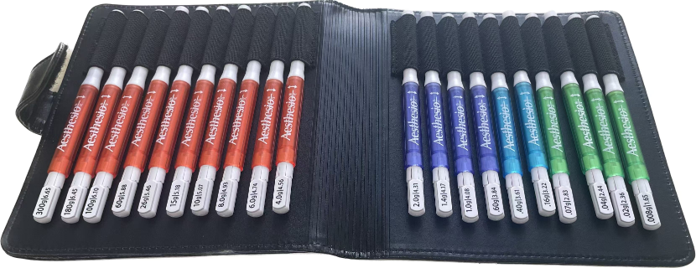

Supplement: Supplementary 1 — Notes S1 to S6 Movies S1 to S4 Figs. S1 to S17 Tables S1 and S2 [file cbsystems.0515.f1.zip › Supplementary Fig. S14.tif]

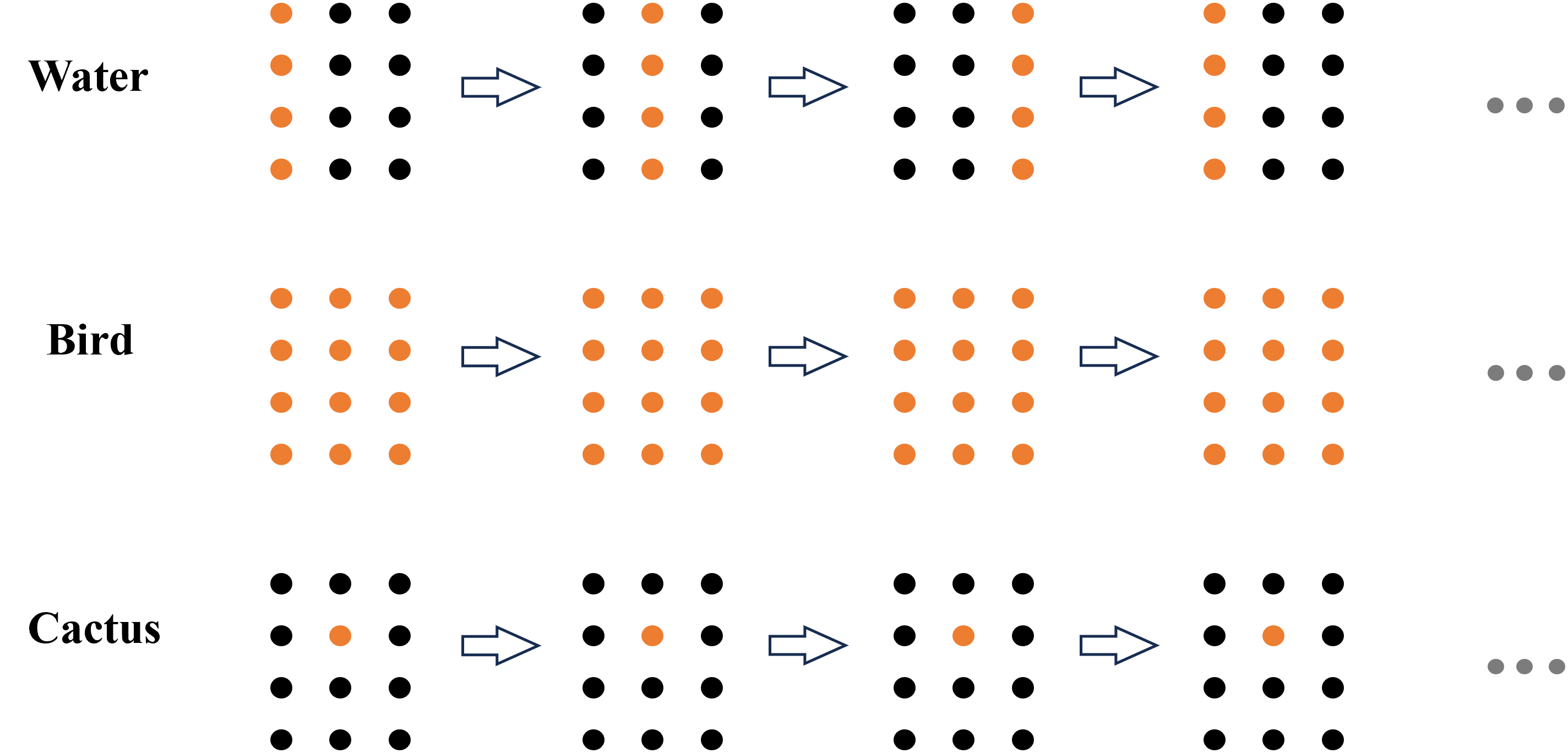

Supplement: Supplementary 1 — Notes S1 to S6 Movies S1 to S4 Figs. S1 to S17 Tables S1 and S2 [file cbsystems.0515.f1.zip › Supplementary Fig. S17.tif]

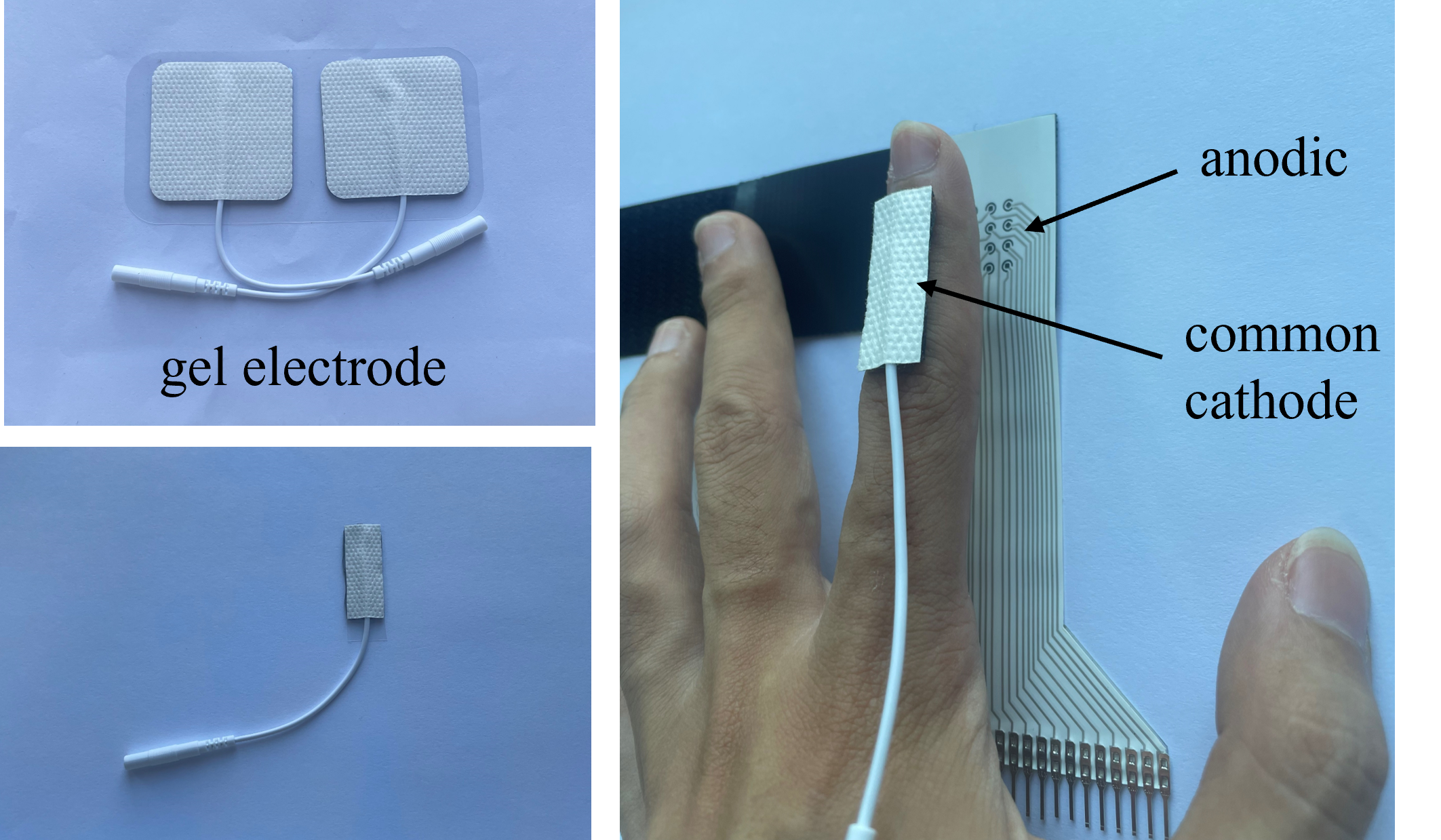

Supplement: Supplementary 1 — Notes S1 to S6 Movies S1 to S4 Figs. S1 to S17 Tables S1 and S2 [file cbsystems.0515.f1.zip › Supplementary Fig. S2.tif]

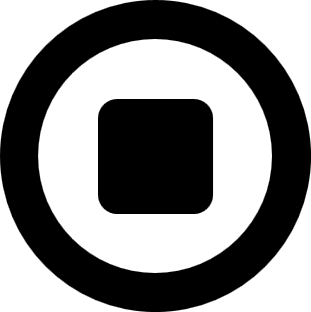

Supplement: Supplementary 1 — Notes S1 to S6 Movies S1 to S4 Figs. S1 to S17 Tables S1 and S2 [file cbsystems.0515.f1.zip › Supplementary Fig. S3.tif]

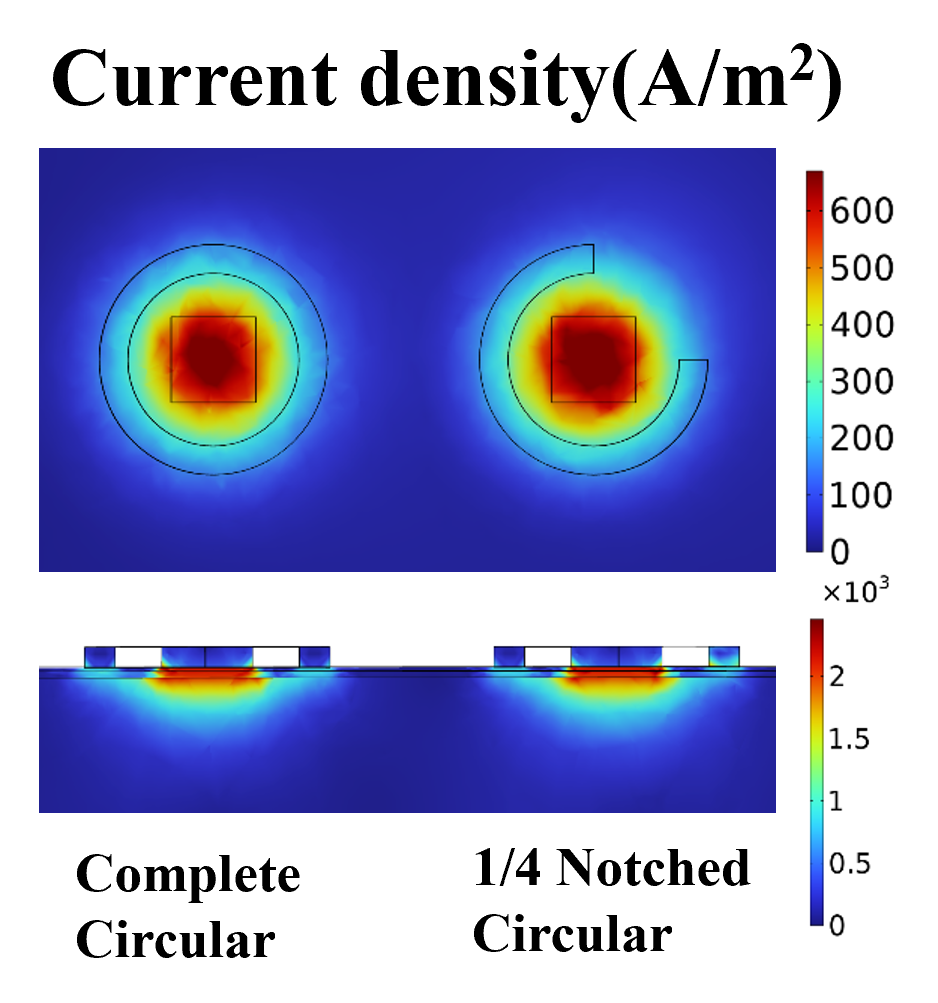

Supplement: Supplementary 1 — Notes S1 to S6 Movies S1 to S4 Figs. S1 to S17 Tables S1 and S2 [file cbsystems.0515.f1.zip › Supplementary Fig. S4.tif]

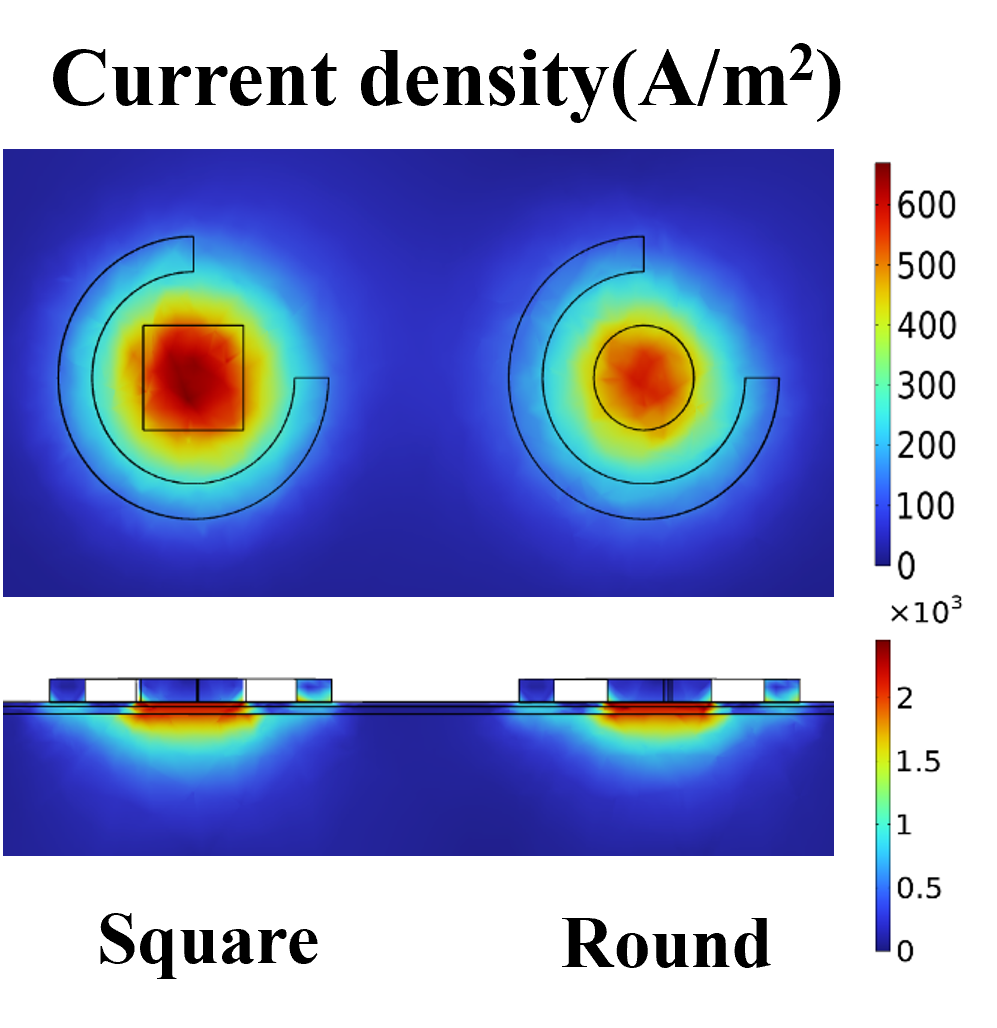

Supplement: Supplementary 1 — Notes S1 to S6 Movies S1 to S4 Figs. S1 to S17 Tables S1 and S2 [file cbsystems.0515.f1.zip › Supplementary Fig. S5.tif]

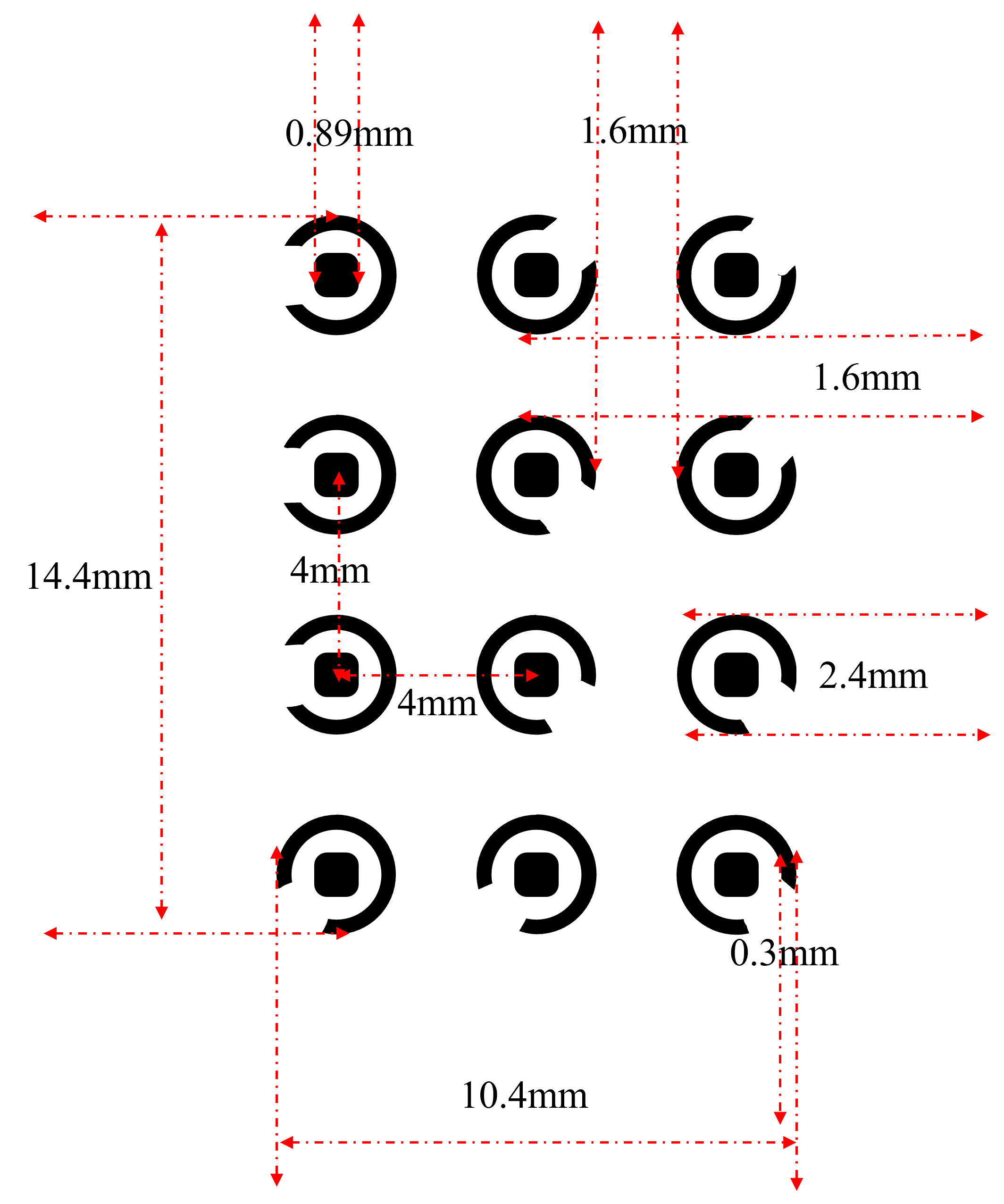

Supplement: Supplementary 1 — Notes S1 to S6 Movies S1 to S4 Figs. S1 to S17 Tables S1 and S2 [file cbsystems.0515.f1.zip › Supplementary Fig. S6.tif]

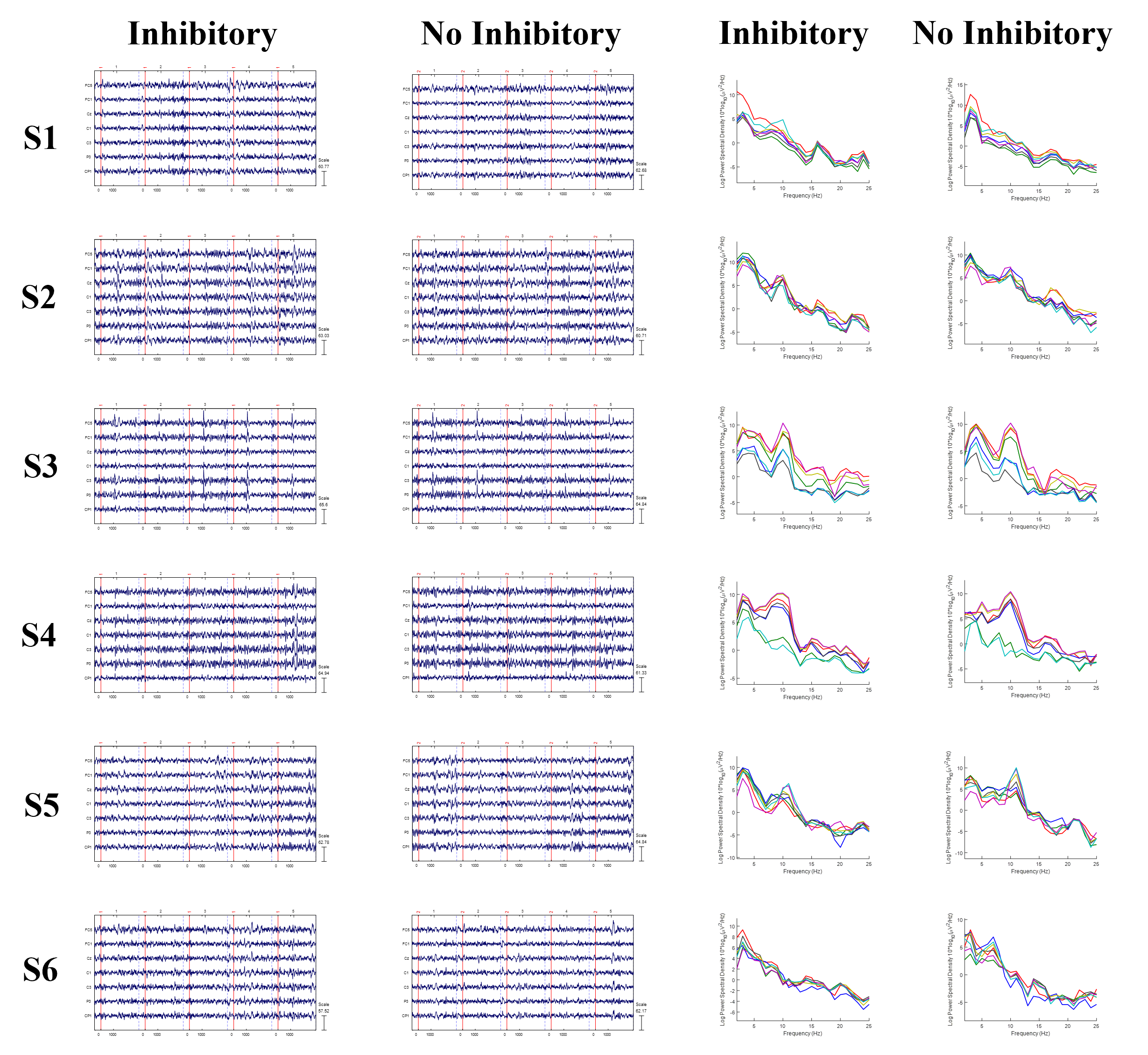

Supplement: Supplementary 1 — Notes S1 to S6 Movies S1 to S4 Figs. S1 to S17 Tables S1 and S2 [file cbsystems.0515.f1.zip › Supplementary Fig. S7.tif]

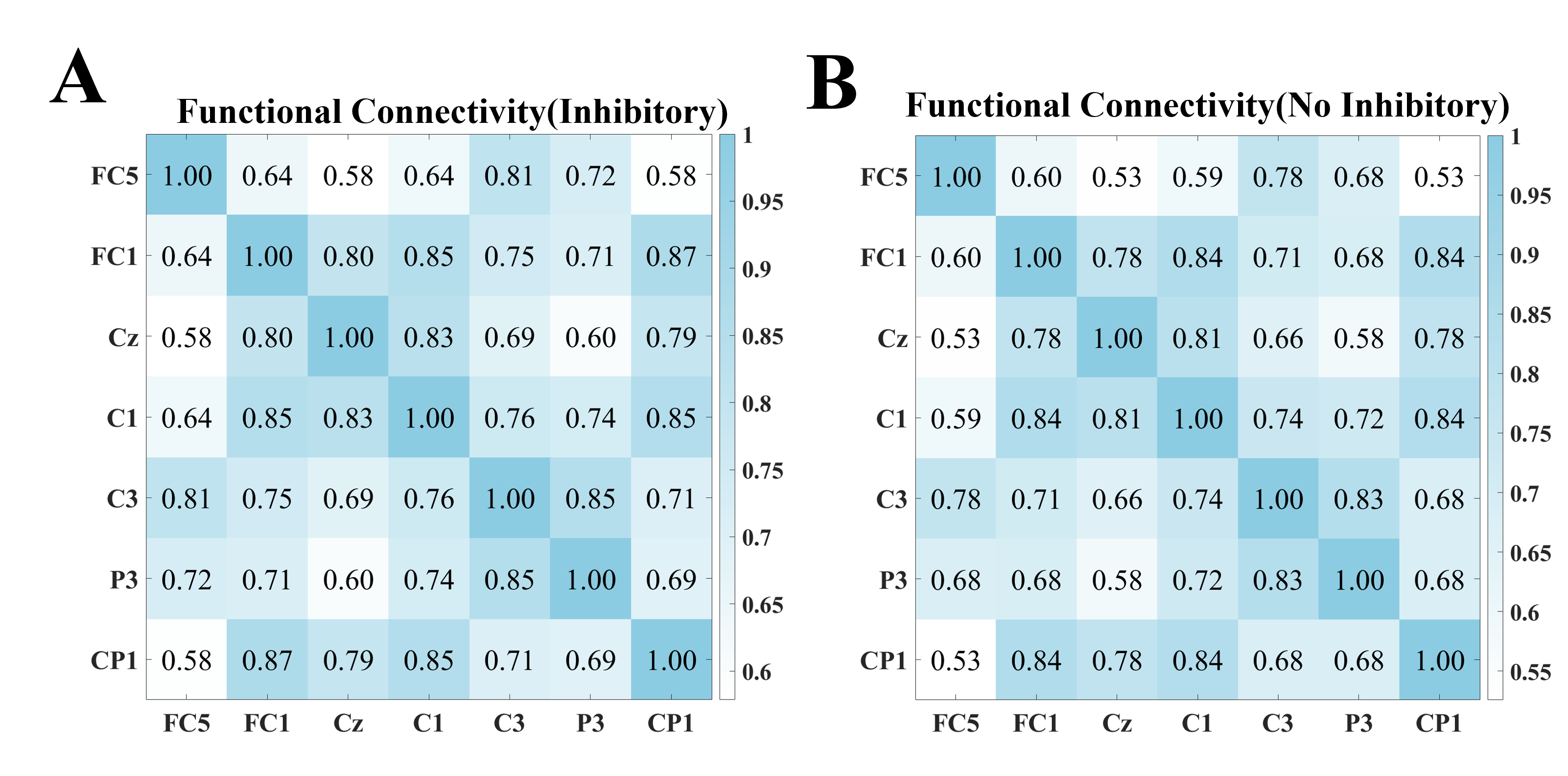

Supplement: Supplementary 1 — Notes S1 to S6 Movies S1 to S4 Figs. S1 to S17 Tables S1 and S2 [file cbsystems.0515.f1.zip › Supplementary Fig. S8.tif]

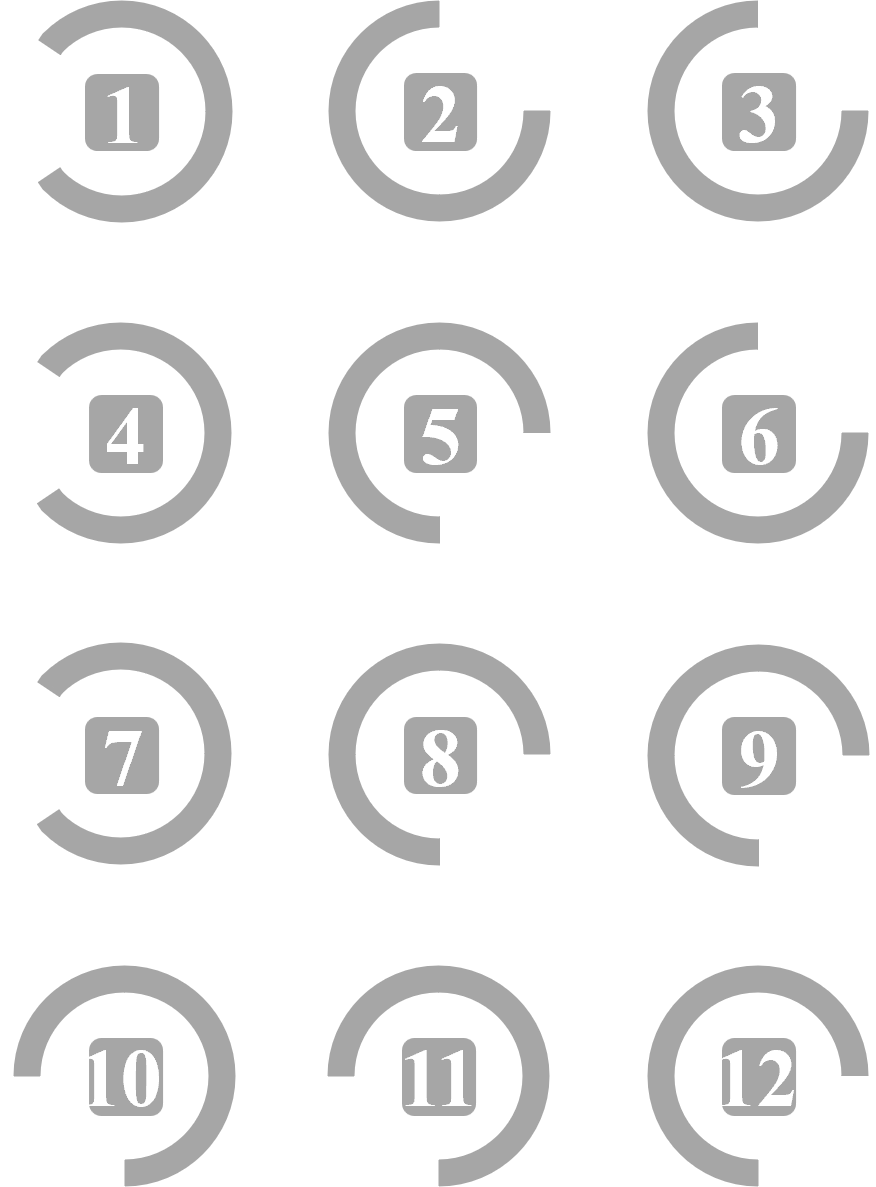

Supplement: Supplementary 1 — Notes S1 to S6 Movies S1 to S4 Figs. S1 to S17 Tables S1 and S2 [file cbsystems.0515.f1.zip › Supplementary Fig. S9.tif]
